# Algorithmic games for full ground references

**DOI:** 10.1007/s10703-017-0292-9

**Published:** 2017-08-09

**Authors:** Andrzej S. Murawski, Nikos Tzevelekos

**Affiliations:** 1 University of Warwick, Coventry, UK; 2 Queen Mary University of London, London, UK

**Keywords:** Program equivalence, Game semantics, Full abstraction, Automata over infinite alphabets

## Abstract

We present a full classification of decidable and undecidable cases for contextual equivalence in a finitary ML-like language equipped with full ground storage (both integers and reference names can be stored). The simplest undecidable type is $\mathsf {unit}\rightarrow \mathsf {unit}\rightarrow \mathsf {unit}$. At the technical level, our results marry game semantics with automata-theoretic techniques developed to handle infinite alphabets. On the automata-theoretic front, we show decidability of the emptiness problem for register pushdown automata extended with fresh-symbol generation.

## Introduction

Mutable variables in which numerical values can be stored for future access and update are the pillar of imperative programming. The memory in which the values are deposited can be allocated statically, typically to coincide with the lifetime of the defining block, or dynamically, on demand, with the potential to persist forever. In order to support memory management, modern programming languages feature mechanisms such as *pointers* or *references*, which allow programmers to access memory via addresses. Languages like C (through int*) or ML (via $\texttt {\small int}\,\texttt {\small ref}\,\texttt {\small ref}$) make it possible to store the addresses themselves, which creates the need for storing references to references etc. We refer to this scenario as *full ground storage*. In this paper we study an ML-like language $\mathsf {GRef}$ with full ground storage, which permits the creation of references to integers as well as references to integer references, and so on.

We concentrate on contextual equivalence^1^ in that setting. Reasoning about program equivalence has been a central topic in programming language semantics since its inception. This is in no small part due to important applications, such as verification problems (equivalence between a given implementation and a model implementation) and compiler optimization (equivalence between the original program and its transform). Specifically, we attack the problem of automated reasoning about our language in a finitary setting, with finite datatypes and with looping instead of recursion, where decidability questions become interesting and the decidability/undecidability frontier can be identified. In particular, it is possible to quantify the impact of higher-order types on decidability, which goes unnoticed in Turing-complete frameworks.

The paper presents a complete classification of cases in which $\mathsf {GRef}$ program equivalence is decidable. The result is phrased in terms of the syntactic shape of types. We write ${\theta _1,\cdots ,\theta _k} \vdash {\theta }$ to refer to the problem of deciding contextual equivalence between two terms $M_1, M_2$ such that ${x_1:\theta _1,\cdots ,x_m:\theta _m} \vdash {M_i:\theta }$ ($i=1,2$). We investigate the problem using a fully abstract game model of $\mathsf {GRef}$.^2^ Such a model can be easily obtained by modifying existing models of more general languages, e.g. by either adding type information to Laird’s model of untyped references [19] or trimming down our own model for general references [24]. The models are *nominal* in that moves may involve elements from an infinite set of *names* to account for reference names. Additionally, each move is equipped with a store whose domain consists of all names that have been revealed (played) thus far and the corresponding values. Note that values of reference types also become part of the domain of the store. This representation grows as the play unfolds and new names are encountered. We shall rely on the model both for decidability and undecidability results. Our work identifies the following undecidable cases as minimal.Obviously, undecidability extends to typing judgments featuring syntactic supertypes of those listed above (for instance, when fourth-order types appear on the left-hand side of the turnstile or types of the shape $\theta _1\rightarrow \theta _2\rightarrow \theta _3$ occur on the right). The remaining cases are summarized by typing judgements in which each of $\theta _1,\cdots , \theta _m$ is generated by the grammar given on the left below, and $\theta$ by the grammar on the right,

$$\begin{aligned} \varTheta _L\; {:}{:}{=}\; \beta \ |\ \varTheta _R\rightarrow \varTheta _L \qquad \qquad \varTheta _R\; {:}{:}{=}\; \beta \ |\ \varTheta _1\rightarrow \beta \end{aligned}$$

where $\beta$ stands any ground type and $\varTheta _1$ is a first-order type, i.e. $\beta \, {:}{:}{=} \mathsf {unit}\ |\ \mathsf {int}\ |\ \mathsf {ref}^i\, \mathsf {int}$ and $\varTheta _1\, {:}{:}{=} \beta \ |\ \beta \rightarrow \varTheta _1$. We shall show that all these cases are in fact decidable. In order to arrive at a decision procedure we rely on effective reducibility to a canonical ($\beta$-normal) form. These forms are then inductively translated into a class of automata over infinite alphabets that represent the associated game semantics. Finally, we show that the representations can be effectively compared for equivalence.

The automata we use are especially designed to read moves-with-stores in a single computational step. They are equipped with a finite set of registers for storing elements from the infinite alphabet (names). Moreover, in a single transition step, the content of a subset of registers can be pushed onto the stack (along with a symbol from the stack alphabet), to be popped back at a later stage. We use visibly pushdown stacks [3], i.e. the alphabet can be partitioned into letters that consistently trigger the same stack actions (push, pop or no-op). Conceptually, the automata extend register pushdown automata [7] with the ability to generate fresh names, as opposed to their existing capability to generate names not currently present in registers. Crucially, we can show that the emptiness problem for the extended machine model remains decidable.

Because the stores used in game-semantic plays can grow unboundedly, one cannot hope to construct the automata in such a way that they will accept the full game semantics of terms. Instead we construct automata that, without loss of generality, will accept plays in which the domains of stores are bounded in size. Each such restricted play can be taken to represent a *set* of real plays compatible with the representation. Compatibility means that values of names omitted in environment-moves (*O*-moves) can be filled in arbitrarily, but values of names omitted in program-moves (*P*-moves) must be the same as in preceding *O*-moves. That is to say, the omissions leading to bounded representation correspond to copy-cat behaviour.

Because we work with representations of plays, we cannot simply use off-the-shelf procedures for checking program equivalence, as the same plays can be represented in different ways: copy-cat behaviour can be modelled explicitly or implicitly via the convention. However, taking advantage of the fact that stacks of two visibly pushdown automata over the same partitioning of the alphabet can be synchronized, we show how to devise another automaton that can run automata corresponding to two terms in parallel and detect inconsistencies in the representations of plays. Exploiting decidability of the associated emptiness problem, we can conclude that $\mathsf {GRef}$ program equivalence in the above-mentioned cases is decidable.

This article is the journal version of [25], with full proofs and rearrangement of the material. Also, we relate our work to what has been done after the conference version was published. We start by introducing the language $\mathsf {GRef}$ in Sect. 2 along with a canonical form result. Then, in Sect. 3 we introduce the game model of $\mathsf {GRef}$ (we refer the interested reader to [28] for a detailed account of the model, which is in effect a restricted version of game models for larger languages presented in [19, 24]). The first main results are obtained in Sect. 4 and concern undecidability of equivalence in specific fragments of $\mathsf {GRef}$ via reductions to queue machine problems. Finally, in Sect. 5 we present a decidability procedure for equivalence in the remaining fragment . The argument implements a reduction to checking non-emptiness in a specific kind of automata over infinite alphabets, the decidability of which is proved in Sect. 5.4.

## $\mathsf {GRef}$

We work with a finitary ML-like language $\mathsf {GRef}$ whose types $\theta$ are generated according to the following grammar.

$$\begin{aligned} \theta \,{:}{:}\,{=} \beta \ |\ \theta \rightarrow \theta \qquad \beta \;{:}{:}{=}\; \mathsf {unit}\ |\ \gamma \qquad \gamma \;{:}{:}{=}\; \mathsf {int}\ |\ \mathsf {ref}\, \gamma \end{aligned}$$

Note that reference types are available for each type of the shape $\gamma$ (full ground storage). The language is best described as the call-by-value $\lambda$-calculus over the ground types $\beta$ augmented with finitely many constants, do-nothing command, case distinction, looping, and reference manipulation (allocation, dereferencing, assignment). The typing rules are:

$$\begin{aligned}&\frac{}{\displaystyle {\varGamma } \vdash {\mathsf {()}:\mathsf {unit}}} \qquad \frac{\displaystyle i\in \{0,\cdots , max \}}{\displaystyle {\varGamma } \vdash {i:\mathsf {int}}}\qquad \frac{\displaystyle (x:\theta )\in \varGamma }{\displaystyle {\varGamma } \vdash {x:\theta }} \qquad \frac{\displaystyle {\varGamma } \vdash {M:\mathsf {int}}\;\;{\varGamma } \vdash {N:\mathsf {unit}}}{\displaystyle {\varGamma } \vdash {\mathsf {while}\,M\,\mathsf {do}\,N:\mathsf {unit}}} \\&\frac{\displaystyle {\varGamma } \vdash {M:\mathsf {int}}\quad {\varGamma } \vdash {N_0:\theta }\quad \cdots \quad {\varGamma } \vdash {N_{ max }:\theta }}{\displaystyle {\varGamma } \vdash {\mathsf {case}(M)[N_0,\cdots ,N_{ max }]:\theta }}\qquad \frac{\displaystyle {\varGamma } \vdash {M:\theta \rightarrow \theta '}\;\;{\varGamma } \vdash {N:\theta }}{\displaystyle {\varGamma } \vdash {MN:\theta '}} \\&\frac{\displaystyle {\varGamma \cup \{x:\theta \}} \vdash {M:\theta '}}{\displaystyle {\varGamma } \vdash {\lambda x^\theta .M:\theta \rightarrow \theta '}}\quad \frac{\displaystyle {\varGamma } \vdash {M:\gamma }}{\displaystyle {\varGamma } \vdash {\displaystyle \mathsf {ref}_{\gamma }(M) : \mathsf {ref}\, \gamma }}\quad \frac{\displaystyle {\varGamma } \vdash {M:\mathsf {ref}\, \gamma }}{\displaystyle {\varGamma } \vdash {!M:\gamma }}\quad \frac{\displaystyle {\varGamma } \vdash {M:\mathsf {ref}\, \gamma }\quad {\varGamma } \vdash {N:\gamma }}{\displaystyle {\varGamma } \vdash {M:=N:\mathsf {unit}}} \end{aligned}$$

In what follows, we write *M*; *N* for the term $$(\lambda z^\theta . N) M$$, where *z* does not occur in *N* and $$\theta $$ matches the type of *M*. $$\mathsf {let}\,x=M\,\mathsf {in}\,N$$ will stand for $$(\lambda x^\theta .N) M$$ in general. The operational semantics of the language can be found in [24, 28]. Note that, assuming $$ max >0$$, reference equality is expressible in the above syntax [29].

### Definition 1

Given ${} \vdash {M:\mathsf {unit}}$, we write $M{\Downarrow }$ if *M* evaluates to (). We say that the term-in-context ${\varGamma } \vdash {M_1:\theta }$ ***approximates*** ${\varGamma } \vdash {M_2:\theta }$ (written ) if  implies  for any context ${C}[-]$ such that . Two terms-in-context are ***equivalent*** if one approximates the other (written ).

We next present a canonical form result for $\mathsf {GRef}$ terms. Its use will become apparent when demonstrating decidability of equivalence in the fragment  of $\mathsf {GRef}$, in Sect. 5. The grammar below defines a notion of canonical form for $\mathsf {GRef}$ terms.

$$\begin{aligned} \mathbb {C}\, {:}{:}\,{=}&()\,\,\,|\,\,\, i\,\,\,|\,\,\, x^{\mathsf {ref}\, \gamma }\,\,\,|\,\,\, \lambda x^{\theta }.\mathbb {C}\,\,\,|\,\,\, \mathsf {case}(x^\mathsf {int})[\mathbb {C},\cdots ,\mathbb {C}] \,\,\,|\,\,\, (\mathsf {while}\,(!x^{\mathsf {ref}\, \mathsf {int}})\,\mathsf {do}\,\mathbb {C});\mathbb {C}\\&|\,\,\, \mathsf {let}\,y^\gamma =\mathsf {!}x^{\mathsf {ref}\, \gamma }\,\mathsf {in}\,\mathbb {C} \,\,\,|\,\,\, (x^{\mathsf {ref}\, \mathsf {int}}:=i);\mathbb {C}\,\,\,|\,\,\, (x^{\mathsf {ref}^2\, \gamma }:=y^{\mathsf {ref}\, \gamma });\mathbb {C}\\&|\,\,\, \mathsf {let}\,x^{\mathsf {ref}\, \mathsf {int}}=\mathsf {ref}(0)\,\mathsf {in}\,\mathbb {C} \,\,\,|\,\,\, \mathsf {let}\,x^{\mathsf {ref}^2\, \gamma }=\mathsf {ref}(y^{\mathsf {ref}\, \gamma })\,\mathsf {in}\,\mathbb {C} \,\,\,|\,\,\, \mathsf {let}\,y= z\, ()\,\mathsf {in}\,\mathbb {C} \\&|\,\,\, \mathsf {let}\,y= z\, i\,\mathsf {in}\,\mathbb {C}\,\,\,|\,\,\, \mathsf {let}\,y= z\, x^{\mathsf {ref}\, \gamma }\,\mathsf {in}\,\mathbb {C} \,\,\,|\,\,\, \mathsf {let}\,y= z\, (\lambda x^\theta .\mathbb {C})\,\mathsf {in}\,\mathbb {C} \end{aligned}$$

### Lemma 2

Let  be an $\mathsf {GRef}$-term. There exists a $\mathsf {GRef}$-term ${\varGamma } \vdash {\mathbb {C}_M:\theta }$ in canonical form, effectively constructible from *M*, such that ${\varGamma } \vdash {M\cong \mathbb {C}_M}$.

## Game semantics

Game semantics views computation as a dialogue between the environment (Opponent, *O*) and the program (Proponent, *P*). We give an overview of the fully abstract game model of $\mathsf {GRef}$ [28]. Let $\mathbb {A}=\biguplus _\gamma \mathbb {A}_{\gamma }$ be a collection of countably infinite sets of *reference names*, or just *names*. The model is constructed using mathematical objects (moves, plays, strategies) that will feature names drawn from $\mathbb {A}$. Although names underpin various elements of our model, their precise nature is irrelevant. Hence, all of our definitions preserve name-invariance, i.e. our objects are (strong) *nominal sets* [11, 32]. Note that we do not need the full power of the theory but mainly the basic notion of name-permutation. For an element *x* belonging to a (nominal) set *X*, we write $\nu (x)$ for its name-support, i.e. the set of names occurring in *x*. Moreover, for any $x,y\in X$, we write $x\sim y$ if *x* and *y* are the same up to a permutation of $\mathbb {A}$. Our model is couched in the Honda-Yoshida style of modelling call-by-value computation [13]. Before we define what it means to play our games, let us introduce the auxiliary concept of an arena. *Q* and *A* are used to distinguish question- and answer-moves respectively.

### Definition 3

An *arena* $A=\langle M_A,I_A,\lambda _A,\vdash _A\rangle$ is given by a set $M_A$ of moves, its subset $I_A$ of initial ones, a labelling function $\lambda _A:M_A\rightarrow \{O,P\}\times \{Q,A\}$ and a justification relation $\vdash _A\; \subseteq M_A\times (M_A{\setminus } I_A)$. These satisfy, for each $m,m'\in M_A$, the conditions:

- $$m\in I_A\implies \lambda _A(m)=(P,A)$$,
- $$m\vdash _A m'\wedge \lambda _A^{QA}(m)=A \implies \lambda _A^{QA}(m')=Q$$,
- and $$m\vdash _A m' \implies \lambda _A^{OP}(m)\not =\lambda _A^{OP}(m')$$.

where we write $\lambda _A^{OP}$ and $\lambda _A^{QA}$ for $\lambda _A$ post-composed with the first and second projections respectively.

We shall use $\mathrm {i}$ to refer to initial moves. Let $\overline{\lambda }_A$ be the *OP*-complement of $\lambda _A$. Given arenas *A*, *B*, the arenas $A\otimes B$ and $A\Rightarrow B$ are constructed as below, where $\bar{I}_A=M_A{\setminus } I_A$, $\bar{\vdash }_A=(\,\vdash _A\upharpoonright \bar{I}_A{}\times \bar{I}_A)$ (and similarly for *B*).

$$\begin{aligned} \begin{aligned} M_{A\Rightarrow B}&= \{\star \}\uplus M_A\uplus M_B&\lambda _{A\Rightarrow B}&= \left[ \,\star \mapsto PA,\, \overline{\lambda }_A[\mathrm {i}_A\mapsto OQ],\, \lambda _B \right] \\ I_{A\Rightarrow B}&= \{\star \}&\vdash _{A\Rightarrow B}&= \left\{ (\star ,\mathrm {i}_A),(\mathrm {i}_A,\mathrm {i}_B)\right\} \, \cup \, \vdash _A\cup \, \vdash _B \\ M_{A\otimes B}&= (I_A\times I_B)\uplus \bar{I}_A\uplus \bar{I}_B&\lambda _{A\otimes B}&= \left[ (\mathrm {i}_A,\mathrm {i}_B)\mapsto PA,\, \lambda _A\upharpoonright \bar{I}_A,\, \lambda _B\upharpoonright \bar{I}_B\right] \\ I_{A\otimes B}&= I_A\times I_B&\vdash _{A\otimes B}&= \left\{ ((\mathrm {i}_A,\mathrm {i}_B),m)\ |\ \mathrm {i}_A\vdash _A m \vee \mathrm {i}_B\vdash _B m\right\} \cup \,\bar{\vdash }_A\cup \,\bar{\vdash }_B \end{aligned} \end{aligned}$$

Let us write [*i*, *j*] for the set $\{i, i+1,\cdots , j\}$. For each type $\theta$ we can define the corresponding arena $\llbracket \theta \rrbracket$.

$$\begin{aligned} \llbracket \mathsf {unit} \rrbracket&= \langle \{\star \},\{\star \},\emptyset ,\emptyset \rangle \qquad \quad \llbracket \mathsf {int} \rrbracket = \langle [0, max ],[0, max ],\emptyset ,\emptyset \rangle \\ \llbracket \mathsf {ref}\, \gamma \rrbracket&= \langle \mathbb {A}_{\gamma },\mathbb {A}_{\gamma },\emptyset ,\emptyset \rangle \qquad \quad \llbracket \theta \rightarrow \theta ' \rrbracket = \llbracket \theta \rrbracket \Rightarrow \llbracket \theta ' \rrbracket \end{aligned}$$

Although types are interpreted by arenas, the actual games will be played in *prearenas*, which are defined in the same way as arenas with the exception that initial moves are O-questions. Given arenas *A*, *B* we define the prearena $A\rightarrow B$ as follows.

$$\begin{aligned} M_{A\rightarrow B}&= M_A\uplus M_B&\lambda _{A\rightarrow B}&= [\overline{\lambda }_A[\mathrm {i}_A\mapsto OQ],\lambda _B] \\ I_{A\rightarrow B}&= I_A&\vdash _{A\rightarrow B}&= \{(\mathrm {i}_A,\mathrm {i}_B)\}\,\cup \,\vdash _A\,\cup \,\vdash _B \end{aligned}$$

A ***store*** is a type-sensitive finite partial function $\varSigma :\mathbb {A}\rightharpoonup [0, max ]\cup \mathbb {A}$ such that $a\in \mathsf {dom}(\varSigma )\cap \mathbb {A}_{\mathsf {int}}$ implies $\varSigma (a)\in [0, max ]$, and $a\in \mathsf {dom}(\varSigma )\cap \mathbb {A}_{\mathsf {ref}\, \gamma }$ implies $\varSigma (a)\in \mathsf {dom}(\varSigma )\cap \mathbb {A}_\gamma$. We write $\mathsf {Sto}$ for the set of all stores. A move-with-store on a (pre)arena *A* is a pair $m^\varSigma$ with $m\in M_A$ and $\varSigma \in \mathsf {Sto}$.

### Definition 4

A *justified sequence* on a prearena *A* is a sequence of moves-with-store on *A* such that, apart from the first move, which must be of the form $\mathrm {i}^\varSigma$ with $\mathrm {i}\in I_A$, every move $n^{\varSigma '}$ in *s* is equipped with a pointer to an earlier move $m^{\varSigma }$ such that $m\vdash _A n$.*m* is then called the justifier of *n*, which is represented as  in drawings.

For each $S\subseteq \mathbb {A}$ and $\varSigma$ we define $\varSigma ^0(S)=S$ and $\varSigma ^{i+1}(S)=\varSigma (\varSigma ^{i}(S))\cap \mathbb {A}$ ($i\ge 0$). Let $\varSigma ^{*}(S)= \bigcup \nolimits _i\varSigma ^{i}(S)$. The set of *available names* of a justified sequence is defined inductively by $\mathsf {Av}(\epsilon )=\emptyset$ and $\mathsf {Av}(s m^\varSigma ) = \varSigma ^{*}(\mathsf {Av}(s)\cup \nu (m))$. The *view* of a justified sequence is defined by:We shall write $s\sqsubseteq s'$ to mean that *s* is a prefix of $s'$.

### Definition 5

Let *A* be a prearena. A justified sequence *s* on *A* is called a ***play***, if it satisfies the conditions below.

- No adjacent moves belong to the same player (*Alternation*).
- The justifier of each answer is the most recent unanswered question (*Bracketing*).
- For any $$s' m^\varSigma \sqsubseteq s$$ with non-empty $$s'$$, the justifier of *m* occurs in $$ view (s')$$ (*Visibility*).
- For any $s'm^\varSigma \sqsubseteq s$, $\mathsf {dom}(\varSigma )=\mathsf {Av}(s'm^\varSigma )$ (*Frugality*).

### Definition 6

A ***strategy*** $\sigma$ on a prearena *A*, written $\sigma :A$, is a set of even-length plays of *A* satisfying:

- If $so^{\varSigma }p^{\varSigma '}\in \sigma$ then $s\in \sigma$ (*Even-prefix closure*).
- If $s\in \sigma$ and $s\sim t$ then $t\in \sigma$ (*Equivariance*).
- If $s_1p_1^{\varSigma _1},s_2p_2^{\varSigma _2}\in \sigma$ and $s_1\sim s_2$ then $s_1p_1^{\varSigma _1}\sim s_2p_2^{\varSigma _2}$ (*Nominal determinacy*).

$\mathsf {GRef}$-terms ${\varGamma } \vdash {M:\theta }$, where $\varGamma =\{x_1:\theta _1,\cdots ,x_n:\theta _n\}$, are interpreted by strategies for the prearena $\llbracket \theta _1 \rrbracket \otimes \cdots \otimes \llbracket \theta _n \rrbracket \rightarrow \llbracket \theta \rrbracket$, which we shall denote by $\llbracket {\varGamma } \vdash {\theta } \rrbracket$. Given a set of plays *X*, let us write  for the set of complete plays in *X*, i.e. those in which each occurrence of a question justifies an answer. The interpretation is then fully abstract in the following sense.

### Proposition 7

([19, 24, 28]) Let ${\varGamma } \vdash {M_1,M_2:\theta }$ be $\mathsf {GRef}$-terms.  if, and only if, . Hence, ${\varGamma } \vdash {M_1\cong M_2}$ if, and only if, .

We shall rely on the result for proving both undecidability and decidability results, by referring to complete plays generated by terms.

### Example 8

The name-generating term ${} \vdash {\lambda x^\mathsf {unit}. \mathsf {ref}(0):\mathsf {unit}\rightarrow \mathsf {ref}\, \mathsf {int}}$ yields complete plays of the shape given below (the corresponding prearena is given on the right). 
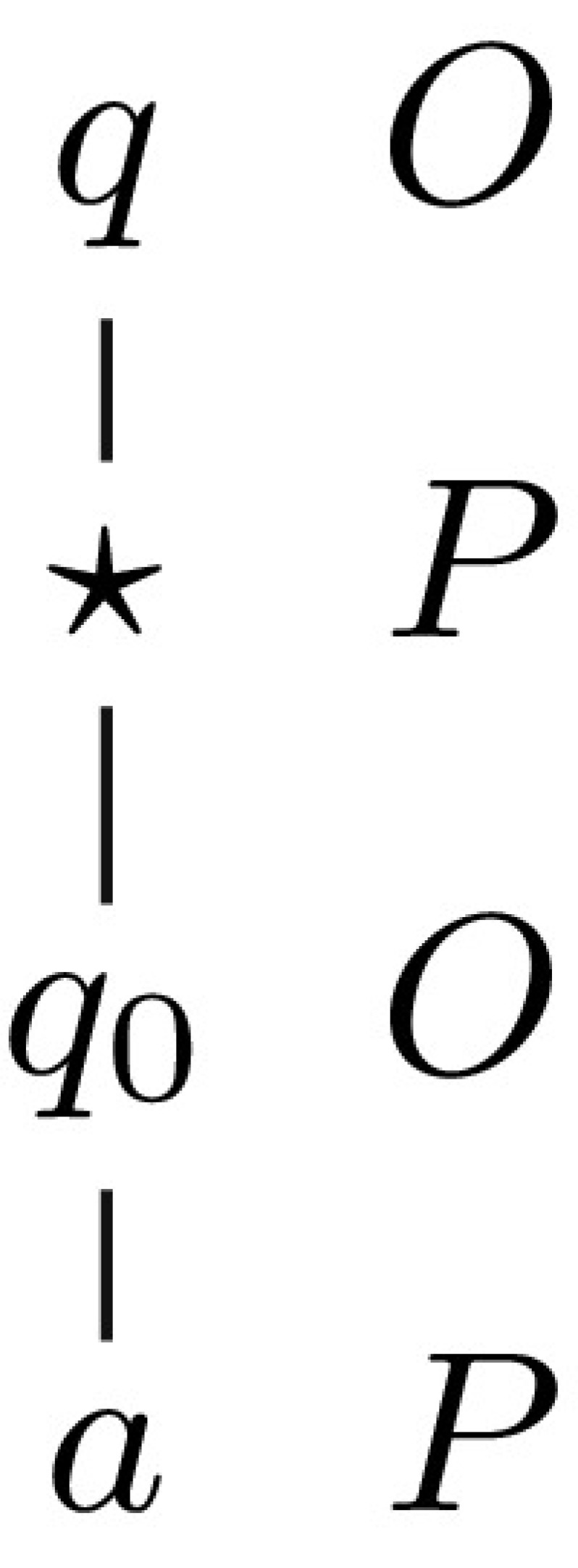
 
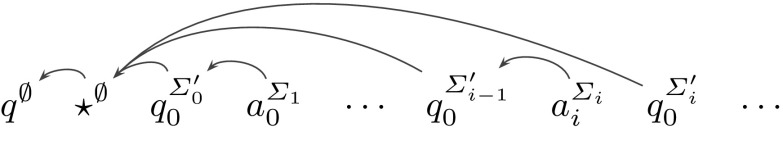
 where $\varSigma _0'=\emptyset$ and, for all $i>0$, $\varSigma _{i}=\varSigma _{i-1}'\cup \{(a_{i},0)\}$, $\mathsf {dom}{(\varSigma _i')}=\mathsf {dom}{(\varSigma _i)}$. Moreover, for any $i\ne j$ we have $a_i\ne a_j$. Note that $\varSigma _i'$ can be different from $\varSigma _i$, i.e. the environment is free to change the values stored at all of the locations that have been revealed to it.

Note that in the above example the sizes of stores keep on growing indefinitely. However, the essence of the strategy is already captured by plays of the shape $q {\star } q_0\, a_0^{(a_0,0)} \cdots q_0\, a_i^{(a_i,0)} q_0\cdots$ under the assumption that, whenever a value is missing from the store of an O-move, it is arbitrary and, for P-moves, it is the same as in the preceding O-move. Next we spell out how a sequence of moves-with-store, not containing enough information to qualify as a play, can be taken to represent proper plays.

### Definition 9

Let $s=m_1^{\varSigma _1}\cdots m_k^{\varSigma _k}$ be a play over ${\varGamma } \vdash {\theta }$ and $t=m_1^{\varTheta _1} \cdots m_k^{\varTheta _k}$ be a sequence of moves-with-store. We say that *s* is an *extension* of *t* if $\varTheta _i\subseteq \varSigma _i$ ($1\le i\le k$) and, for any $1\le i\le \lfloor {k/2}\rfloor$, if $a\in \mathsf {dom}{(\varSigma _{2i})}{\setminus } \mathsf {dom}{(\varTheta _{2i})}$ then $\varSigma _{2i}(a)=\varSigma _{2i-1}(a)$. We write  for the set of all extensions of *t*.

Because we cannot hope to encode plays with unbounded stores through automata, our decidability results will be based on representations of plays that capture strategies via extensions.

## Undecidability arguments

We begin with undecidable cases. Our argument will rely on queue machines, which are finite-state devices equipped with a queue.

### Definition 10

Let $\mathcal {A}$ be a finite alphabet. A queue machine over $\mathcal {A}$ is specified by $\langle Q, Q_E, Q_D, init , \delta _E, \delta _D \rangle$, where *Q* is a finite set of states such that $Q=Q_E\uplus Q_D$, $init \in Q_E$ is the initial state, $\delta _E:Q_E \rightarrow Q\times \mathcal {A}$ is the enqueuing function, whereas $\delta _D: Q_D\times \mathcal {A}\rightarrow Q$ is the dequeuing function.

A queue machine starts at state $init$ with an empty queue. Whenever it reaches a state $q\in Q_E$, it will progress to the state $\pi _1 (\delta _E(q))$ and $\pi _2 (\delta _E(q))$ will be added to the associated queue, where $\pi _1, \pi _2$ are the first and second projections respectively. If the machine reaches a state $q\in Q_D$ and its queue is empty, the machine is said to *halt*. Otherwise, it moves to the state $\delta _D (q,x)$, where *x* is the symbol at the head of the associated queue, which is then removed from the queue. The halting problem for queue machines is well known to be undecidable (e.g. [17]). By encoding computation histories of queue machines as plays generated by $\mathsf {GRef}$ terms we next show that the equivalence problem for $\mathsf {GRef}$ terms must be undecidable. Note that this entails undecidability of the associated notion of term approximation.

### Theorem 11

The contextual equivalence problem is undecidable in the following cases (even in absence of looping).

- $${} \vdash {M_1\cong M_2:\mathsf {unit}\rightarrow \mathsf {unit}\rightarrow \mathsf {unit}}$$
- $${f:(\mathsf {unit}\rightarrow \mathsf {unit}\rightarrow \mathsf {unit})\rightarrow \mathsf {unit}} \vdash {M_1\cong M_2:\mathsf {unit}}$$
- $${f:(((\mathsf {unit}\rightarrow \mathsf {unit})\rightarrow \mathsf {unit})\rightarrow \mathsf {unit})\rightarrow \mathsf {unit}} \vdash {M_1\cong M_2:\mathsf {unit}}$$
- $${} \vdash {M_1\cong M_2: ((\mathsf {unit}\rightarrow \mathsf {unit})\rightarrow \mathsf {unit})\rightarrow \mathsf {unit}}$$

In the following we prove undecidability in each of the cases of Theorem 11.

${} \vdash {{\mathsf {unit}\rightarrow \mathsf {unit}\rightarrow \mathsf {unit}}}$: We first sketch the argument. The arena used to interpret closed terms of type $\mathsf {unit}\rightarrow \mathsf {unit}\rightarrow \mathsf {unit}$ has the shape given on the right.

We are going to use plays from the arena to represent sequences of queue operations. Enqueuing will be represented by segments of the form $q_0 \star _0$, whereas $q_1 \star _1$ will be used to represent dequeuing. Additionally, in the latter case $q_1$ will be justified by $\star _0$ belonging to the segment representing the enqueuing of the element that is now being dequeued. For instance, the sequence *EEDEDE*, in which *E*, *D* stand for enqueing and dequeing respectively, will be represented as follows.

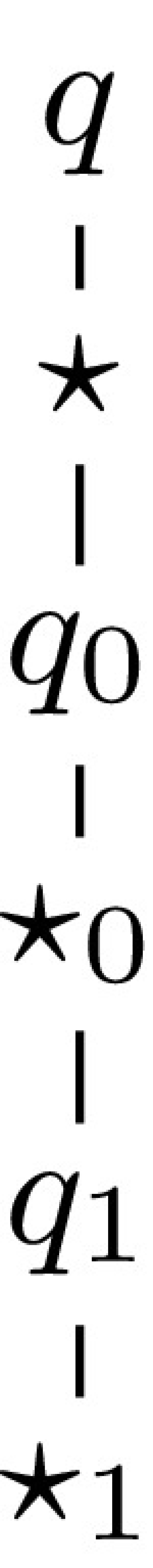

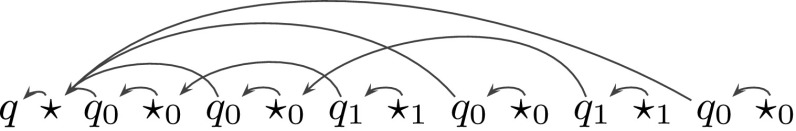





Note that all such plays are complete. Given a queue machine $\mathbb Q$, let us write $\mathsf {hist}(\mathbb Q)$ for the (prefix-closed) subset of $(E\uplus D)^*$ corresponding to all sequences of queue operations performed by $\mathbb Q$. Note that $\mathsf {hist}(\mathbb Q)$ is finite if and only if $\mathbb Q$ halts. Additionally, define $\mathsf {hist}^-(\mathbb Q)$ to be $\mathsf {hist}(\mathbb Q)$ from which the longest sequence is removed (if $\mathsf {hist}(\mathbb Q)$ is infinite and the sequence in question does not exist we set $\mathsf {hist}^-(\mathbb Q)=\mathsf {hist}(\mathbb Q)$). Note that the sequence corresponds to a terminating run and necessarily ends in *D*.

### Lemma 12

Let $\mathbb Q$ be a queue machine. There exist $\mathsf {GRef}$ terms ${} \vdash {M,M^-:\mathsf {unit}\rightarrow \mathsf {unit}\rightarrow }{\mathsf {unit}}$ such that ,  represent $\mathsf {hist}(\mathbb Q)$, $\mathsf {hist}^-(\mathbb Q)$ respectively.

**Fig. 1:**
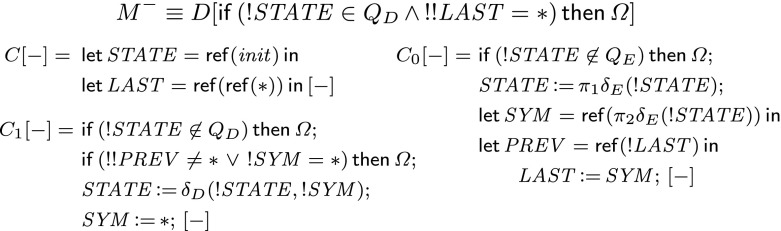
Simulating a queue machine in $\vdash \mathsf {unit}\rightarrow \mathsf {unit}\rightarrow \mathsf {unit}$. The variable $STATE :\mathsf {ref}\, \mathsf {int}$ contains the current state of the machine. The queue is encoded as a backwards-connected list with elements $( PREV , SYM ):\mathsf {ref}^2\, \mathsf {int}\times \mathsf {ref}\, \mathsf {int}$, with last-element pointer $LAST :\mathsf {ref}^2\, \mathsf {int}$. Enqueuing adds a new last element while dequeuing sets the first non-$*$ symbol of the list to $*$

### Proof

WLOG we shall assume that *Q* can be fitted into $\mathsf {int}$ (otherwise, we could use a fixed number of variables to achieve the desired storage capacity). Let $D[-]\equiv C[\lambda x.C_0[\lambda y. C_1 [-]]]$, where $C[-], C_0[-], C_1[-]$ are given in Fig. 1 ($*$ is a special symbol not in the queue alphabet and $\varOmega$ is a canonical divergent term). $C_0[-]$ and $C_1[-]$ handle enqueuing and dequeuing respectively. We take

$$\begin{aligned} M^-\equiv D[\mathsf {if}\,(! STATE \in Q_D\, \wedge \, !! LAST =*)\,\mathsf {then}\,\varOmega ] \end{aligned}$$

and $$M\equiv D[()]$$.

Note that there are only three moves that *O* can play: *q*, $q_0$ and $q_1$. After the initial *q*, *P* must follow with $\star$ thanks to $C[-]$, which will not cause divergence. Note that it declares the variable $STATE$ (initialized to $init$), whose scope spans over the whole term and which will be updated at each step to mimic the state of $\mathbb Q$. After *q* is played, it can never be played again, but *O* can still play $q_0$ or $q_1$. These are handled by $C_0[-]$ and $C_1[-]$ respectively.

- If *O* plays $q_0$ when $\mathbb Q$ is not able to enqueue, *P* will not respond. This is caused by the condition $! STATE \not \in Q_E$ in $C_0[-]$. However, if $\mathbb Q$ is in enqueuing mode, *local* references $SYM$ and $PREV$ will be created. $SYM$ is initialized to the symbol that $\mathbb Q$ will add to the queue. $PREV$ contains the name of the reference cell in which the previously enqueued symbol was stored (as soon as the symbol is dequeued, the value stored in that cell will be set to $*$).
  The “global” reference $LAST$ (of type $\mathsf {ref}(\mathsf {ref}\,\mathsf {int})$) is used to pass the name from one $q_0 \star _0$ segment to the next. Hence, the current value of $SYM$ is written to $LAST$ as soon as the previous value of $LAST$ got recorded in $PREV$. The assignment is followed by the value $\lambda y.C_3[-]$, so *P* will respond with $\star _0$.
- If *O* plays $q_1$ in an enqueuing state, *P* will not respond due to the $! STATE \not \in Q_D$ check in $C_1[-]$. Furthermore, *P* will not reply when
  - $q_1$ is justified by $\star _0$ from a block corresponding to an element that has already been taken off the queue ($! SYM =*$);
  - $q_1$ is justified by $\star _0$ from a block corresponding to elements that are still present in the queue, but do not occur at its head ($!! PREV \ne *$).
  Otherwise (i.e. if *O* plays $q_1$ and justifies it with $\star _0$ from the $q_0 \star _0$ corresponding to the least recent symbol that has not been dequeued) $STATE$ will be updated and $SYM$ will be set to $*$ to record the access. The strategy corresponding to *M* will then reply with $\star _0$ (because of ()). The one associated with $M^-$ will do the same, unless $\mathbb Q$ is about to halt. This is thanks to the $(! STATE \in Q_D\, \wedge \, !! LAST =*)$ condition, which checks whether $\mathbb Q$ is about to dequeue ($! STATE \in Q_D$) the empty queue ($!! LAST =*$).

Hence, *M* and $M^-$ both represent the behaviour of $\mathbb Q$, except that, if $\mathbb Q$ halts, the strategy corresponding to $M_1$ will not generate the last $q_1 \star _1$ segment corresponding to the last dequeuing operation. Consequently, $M_0$ and $M_1$ corresponds to $\mathsf {hist}(\mathbb Q)$ and $\mathsf {hist}^-(\mathbb Q)$ respectively. $\square$

Observe that $\mathsf {hist}(\mathbb Q)=\mathsf {hist}^-(\mathbb Q)$ exactly when $\mathbb Q$ does not halt. Consequently, the problem of deciding $\mathsf {hist}(\mathbb Q)=\mathsf {hist}^-(\mathbb Q)$ is undecidable. Thus, via Proposition 7, we can conclude that program equivalence is undecidable for closed terms of type $\mathsf {unit}\rightarrow \mathsf {unit}\rightarrow \mathsf {unit}$. The remaining cases are discussed below. ${(\mathsf {unit}\rightarrow \mathsf {unit}\rightarrow \mathsf {unit})\rightarrow \mathsf {unit}} \vdash {\mathsf {unit}}$: The arena at hand has the following shape. As before, we use $q_0 \star _0$ and $q_1\star _1$ to represent enqueuing and dequeuing respectively. They will be preceded by a single segment $q q'$. Note that this means that no complete plays will arise until *q* is answered. We shall arrange for this to happen only when the whole terminating run (if any) has been represented.

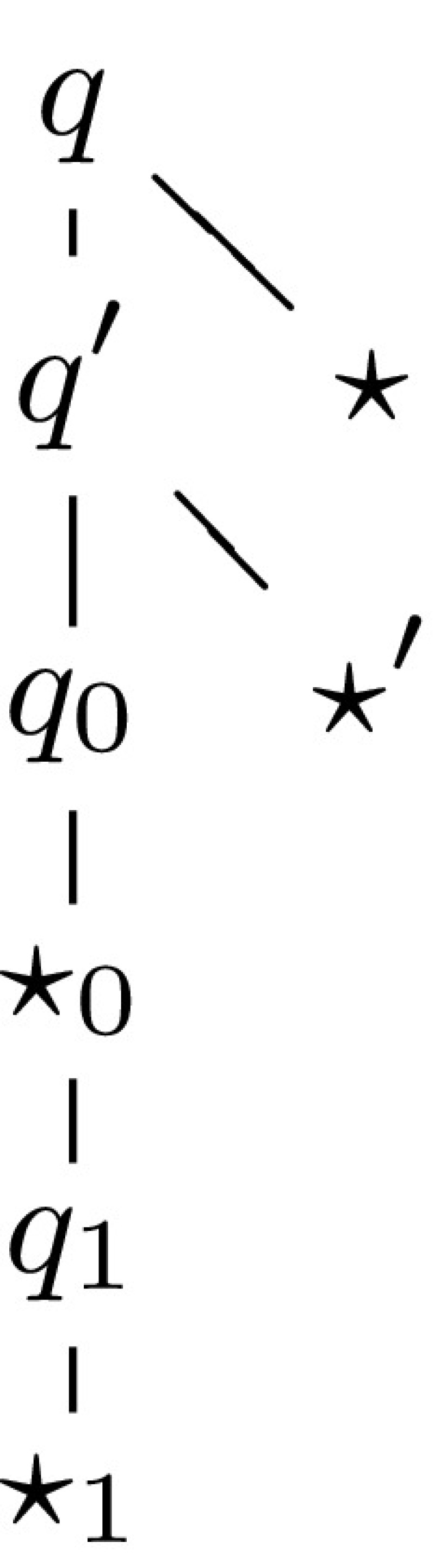



### Lemma 13

Let $\mathbb Q$ be a queue machine. Then there exists a term

$$\begin{aligned} {f: (\mathsf {unit}\rightarrow \mathsf {unit}\rightarrow \mathsf {unit})\rightarrow \mathsf {unit}} \vdash {M:\mathsf {unit}} \end{aligned}$$

such that  if and only $\mathbb Q$ does not halt.

### Proof

Reusing $C[-], C_0[-], C_1[-]$ from the previous case, we take *M* to be $C[ f (\lambda x. C_0 [\lambda y. C_1[()]]); test ]$, where $test$ stands for $\mathsf {if}\,(! STATE \in Q_D\, \wedge \, !! LAST =*)\,\mathsf {then}\,()\,\mathsf {else}\,\varOmega$. The last condition $(! STATE \in Q_D\, \wedge \, !! LAST =*)$ means that whenever *O* plays $\star '$, *P* will not respond unless $\mathbb Q$ terminates and the terminating run has been wholly represented in the play. The argument showing that *M* represents $\mathbb Q$ is analogous to that for Lemma 12. $\square$

### Proposition 14

It is undecidable whether a given term ${f:(\mathsf {unit}\rightarrow \mathsf {unit}\rightarrow \mathsf {unit})\rightarrow \mathsf {unit}} \vdash {M:\mathsf {unit}}$ is equivalent to ().

${(((\mathsf {unit}\rightarrow \mathsf {unit})\rightarrow \mathsf {unit})\rightarrow \mathsf {unit})\rightarrow \mathsf {unit}} \vdash {\mathsf {unit}}$: The corresponding arena has the shape given on the right. Our representation scheme in this case will start off with $q q'$, enqueuing will be interpreted by $q_0 q_1$ and dequeuing by $q_2\star _2$, where $q_2$ is justified by $q_1$ corresponding to the element being dequeued. Note that sequences of this kind are not complete plays, because $q, q', q_0, q_1$ will remain unanswered. Hence, in the term construction it will not be possible to answer them until a terminating run has been fully simulated. Then *O*’s $\star _1$ will trigger *P*’s $\star _0$, and $\star '$ will trigger $\star$.

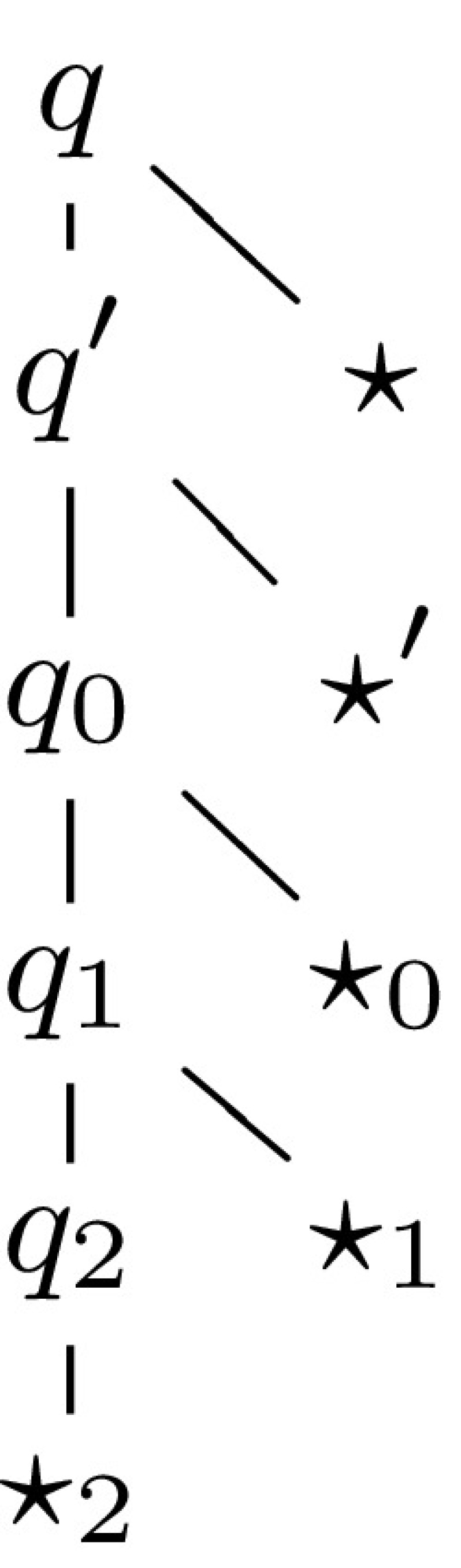



### Lemma 15

Let $\mathbb Q$ be a queue machine. Then there exists a term

$$\begin{aligned} {f: (((\mathsf {unit}\rightarrow \mathsf {unit})\rightarrow \mathsf {unit})\rightarrow \mathsf {unit})\rightarrow \mathsf {unit}} \vdash {M_\mathbb Q:\mathsf {unit}} \end{aligned}$$

such that  if and only $\mathbb Q$ does not halt.

### Proof

Take $M_\mathbb Q$ to be $C[f(\lambda g. C_0[g(\lambda h. C_1[()] )]; test )]; test$. The $test$ phrases block *P* from answering $\star _1$ or $\star '$ prematurely. $\square$

### Proposition 16

The problem of deciding whether a given term

$$\begin{aligned} {f: (((\mathsf {unit}\rightarrow \mathsf {unit})\rightarrow \mathsf {unit})\rightarrow \mathsf {unit})\rightarrow \mathsf {unit}} \vdash {M:\mathsf {unit}} \end{aligned}$$

is equivalent to () is undecidable.

${} \vdash {((\mathsf {unit}\rightarrow \mathsf {unit})\rightarrow \mathsf {unit})\rightarrow \mathsf {unit}}$: The corresponding arena has the shape given on the right. Our representation scheme in this case will start off with $q \star$, enqueuing will be interpreted by $q_0 q_1$ and dequeuing by $q_2\star _2$, where $q_2$ is justified by $q_1$ corresponding to the element being dequeued. Note that, apart from $q\star$, sequences of this kind are not complete plays, because $q_0, q_1$ will remain unanswered. Hence, in the term construction it will not be possible to answer them until a terminating run has been fully simulated. Then *O*’s $\star _1$ will trigger *P*’s $\star _0$.

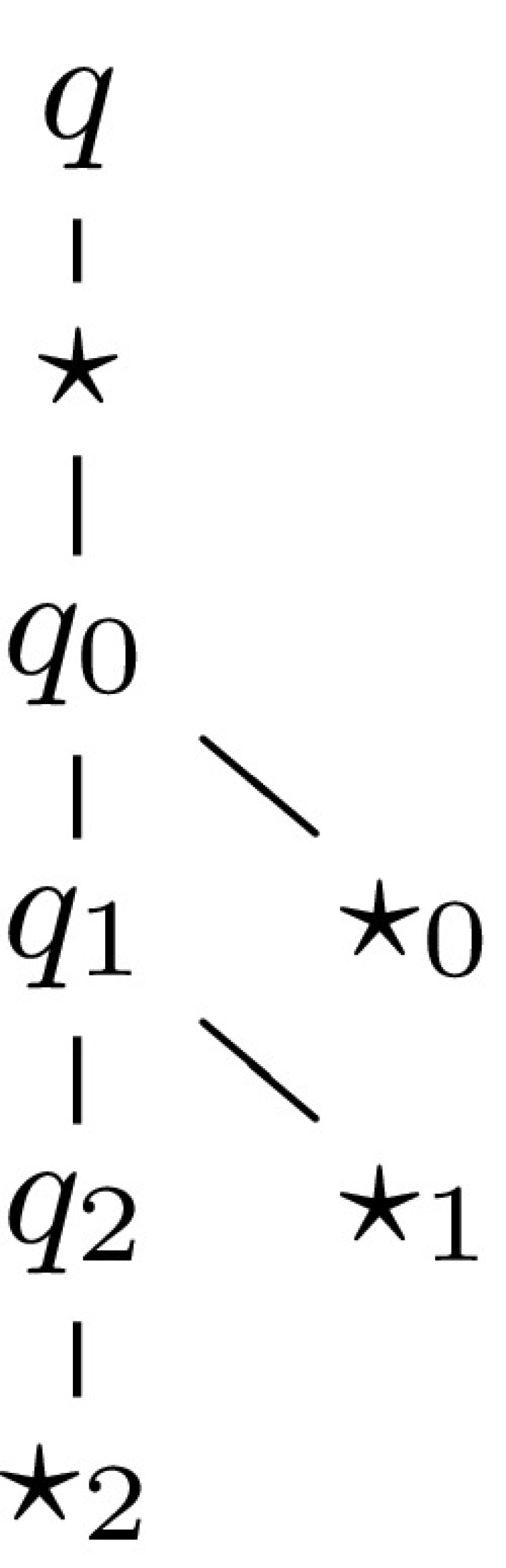



### Lemma 17

Let $\mathbb Q$ be a queue machine. Then there exists a term ${} \vdash {M_\mathbb Q}: ((\mathsf {unit}\rightarrow \mathsf {unit})\rightarrow \mathsf {unit})\rightarrow \mathsf {unit}$ such that  if and only $\mathbb Q$ does not halt.

### Proof

Take $M_\mathbb Q$ to be $C[\lambda f^{(\mathsf {unit}\rightarrow \mathsf {unit})\rightarrow \mathsf {unit}}.C_0[f (\lambda g^\mathsf {unit}. C_1[()]); test ]]$. The $test$ phrases block *P* from answering $\star _1$ prematurely. $\square$

### Proposition 18

The problem of deciding whether a given term ${} \vdash {M}:((\mathsf {unit}\rightarrow \mathsf {unit})\rightarrow \mathsf {unit})\rightarrow \mathsf {unit}$ is equivalent to $\lambda f^{(\mathsf {unit}\rightarrow \mathsf {unit})\rightarrow \mathsf {unit}}.\varOmega$ is undecidable.

## Decidability

We now focus on a fragment of $\mathsf {GRef}$, called , that comprises all types that do *not* fall under the undecidable cases identified earlier.

### Definition 19

Suppose $\varGamma ={x_1:\theta _1,\cdots ,x_m:\theta _m}$. The term-in-context ${\varGamma } \vdash {M:\theta }$ belongs to  provided $\theta _1,\cdots , \theta _m$ can be generated from $\varTheta _L$ and $\theta$ is generated from $\varTheta _R$, where $\varTheta _L{:}{:}{=} \beta \ |\ \varTheta _R\rightarrow \varTheta _L$,   $\varTheta _R{:}{:}{=} \beta \ |\ \varTheta _1\rightarrow \beta$ and $\varTheta _1{:}{:}{=} \beta \ |\ \beta \rightarrow \varTheta _1$.

Put otherwise, we focus on sequents of the form:

$$\begin{aligned} \varTheta _R\rightarrow \cdots \rightarrow \varTheta _R\rightarrow \beta \vdash \varTheta _R \end{aligned}$$

where $$\varTheta _R = (\beta \rightarrow \cdots \rightarrow \beta )\rightarrow \beta $$.

Each type $\theta$ can be written in the form $\theta =\theta _n\rightarrow \ldots \rightarrow \theta _1\rightarrow \beta$, for types $\theta _1,\dots ,\theta _n$ and base type $\beta$. For brevity, we shall write $\theta =(\theta _n,\dots ,\theta _1,\beta )$. We call *n* the *arity* of $\theta$ and denote it by $ar (\theta )$.

### Definition 20

For every type $\theta$ let us define the associated set of labels $\mathcal {L}_{\theta }$ as follows:

$$\begin{aligned} \mathcal {L}_{\mathsf {unit}}&=\{\star \}&\mathcal {L}_{\mathsf {ref}\, \gamma }&=\mathbb {A}_\gamma \\ \mathcal {L}_{\mathsf {int}}&=\{0,\cdots , max \}&\mathcal {L}_{\theta \rightarrow \theta '}&=\{\star \} \end{aligned}$$

We shall write $\mathcal {L}_{}$ for the set of all labels.

Let us fix notation for referring to moves that are available in arenas corresponding to  typing judgments: each move can be viewed as a pair $(l,t)$ subject to consistency constraints induced by the subtypes which contribute them, e.g. the label corresponding to a tag related to $\mathsf {int}$ must be a number from $[0, max ]$.

More precisely, given ${\varGamma } \vdash {M:\theta }$, $l\in \mathcal {L}_{}$ and some symbol *t* (determined below), we shall say that the pair $(l,t)$ is *consistent* if the following conditions are satisfied. Below we assume that $(x:\theta ')\in \varGamma$ and $\theta '\equiv (\theta _m,\cdots ,\theta _1,\beta )$.

- If $$t=\mathsf {r}_{\downarrow }$$ then $$l\in \mathcal {L}_{\theta }$$.
- If $$t=\mathsf {c}^x_i$$ then $$l\in \mathcal {L}_{\theta _i}$$.
- If $$t=\mathsf {r}^x_i$$ then $$l\in \mathcal {L}_{(\theta _{i-1},\dots ,\theta _{1},\beta )}$$.
- If $t=\mathsf {c}^x_{j,i}$ then $l\in \mathcal {L}_{\theta _{j,i}}$, where $\theta _j\equiv \theta _{j,0}\rightarrow \beta$ and $\theta _{j,0}\equiv (\theta _{j,k},\cdots ,\theta _{j,1},\beta ')$.
- If $t=\mathsf {r}^x_{j,i}$ then $l\in \mathcal {L}_{\theta _{j,i}}$, where $\theta _j\equiv (\beta _k,\cdots ,\beta _1,\beta )\rightarrow \beta _{0}$, $\theta _{j,0}\equiv \beta _{0}$ and $\theta _{j,i}\equiv (\beta _{i-1},\cdots ,\beta _1,\beta )$ for $i>0$.
- If $t=\mathsf {c}_i$ then $l\in \mathcal {L}_{\theta _i}$, where $\theta \equiv \theta _0\rightarrow \beta$ and $\theta _0\equiv (\theta _n,\cdots ,\theta _1,\beta ')$.
- If $t=\mathsf {r}_i$ then $l\in \mathcal {L}_{\theta _i}$, where $\theta \equiv (\beta _n,\cdots ,\beta _1,\beta )\rightarrow \beta _0$, $\theta _0\equiv \beta _0$ and $\theta _i\equiv (\beta _{i-1},\cdots ,\beta _1,\beta )$ for $i>0$.

Thus, consistent pairs (*t*, *l*) uniquely specify moves of $\llbracket \varGamma \vdash \theta \rrbracket$. The tag *t* specifies the position of the move within the arena, while *l* determines its actual value. We shall write $\mathbb {T}$ to refer to the set of tags.

In order to show decidability we first translate  terms into automata that represent their game semantics. A corollary of Lemma 2 is that any  term can be effectively converted to an equivalent term in canonical shape.

$$\begin{aligned}\begin{array}{ll} \mathbb {C}\, {:}{:}{=} &{} ()\,\,\,|\,\,\, i\,\,\,|\,\,\, x^{\mathsf {ref}\, \gamma }\,\,\,|\,\,\, \lambda x^{\varTheta _1}.\mathbb {C}\,\,\,|\,\,\, \mathsf {case}(x^\mathsf {int})[\mathbb {C},\cdots ,\mathbb {C}] \,\,\,|\,\,\, (\mathsf {while}\,(!x^{\mathsf {ref}\, \mathsf {int}})\,\mathsf {do}\,\mathbb {C});\mathbb {C}\\ &{} |\,\,\, \mathsf {let}\,y^\gamma =\mathsf {!}x^{\mathsf {ref}\, \gamma }\,\mathsf {in}\,\mathbb {C} \,\,\,|\,\,\, (x^{\mathsf {ref}\, \mathsf {int}}:=i);\mathbb {C}\,\,\,|\,\,\, (x^{\mathsf {ref}^2\, \gamma }:=y^{\mathsf {ref}\, \gamma });\mathbb {C}\\ &{} |\,\,\, \mathsf {let}\,x^{\mathsf {ref}\, \mathsf {int}}=\mathsf {ref}(0)\,\mathsf {in}\,\mathbb {C} \,\,\,|\,\,\, \mathsf {let}\,x^{\mathsf {ref}^2\, \gamma }=\mathsf {ref}(y^{\mathsf {ref}\, \gamma })\,\mathsf {in}\,\mathbb {C} \,\,\,|\,\,\, \mathsf {let}\,y^{\varTheta _L}= z\, ()\,\mathsf {in}\,\mathbb {C} \\ &{} |\,\,\, \mathsf {let}\,y^{\varTheta _L}= z\, i\,\mathsf {in}\,\mathbb {C}\,\,\,|\,\,\, \mathsf {let}\,y^{\varTheta _L}= z\, x^{\mathsf {ref}\, \gamma }\,\mathsf {in}\,\mathbb {C} \,\,\,|\,\,\, \mathsf {let}\,y^{\varTheta _L}= z\, (\lambda x^{\varTheta _1}.\mathbb {C})\,\mathsf {in}\,\mathbb {C} \end{array} \end{aligned}$$

Consequently, it suffices to show that program equivalence between terms in canonical form is decidable. Accordingly, in what follows, we focus exclusively on translating terms in canonical shape.

We next introduce a class of automata (over an infinite input alphabet) which will be the target of our translation from canonical forms of .

### A class of automata

To enable a finite specification of our automata and to describe their semantics we introduce the following definitions. Recall that $\mathbb {A}$ is the set of names, partitioned as:

$$\begin{aligned} \mathbb {A}=\biguplus \nolimits _{\gamma }\mathbb {A}_\gamma \end{aligned}$$

Let $\mathbb {C}=\{\star ,0,\cdots , max \}$ be the set of *constants*. Let us also fix natural numbers $n_{\mathsf {r}},n$ with $n_{\mathsf {r}}\le n$, a finite set $\mathbb {C}_{\mathsf {stack}}$ of *stack symbols* and a finite set $\mathbb {T}$ of *tags*, partitioned into push tags, pop tags and no-op tags:

$$\begin{aligned} \mathbb {T}=\mathbb {T}_{\mathsf {push}}\uplus \mathbb {T}_{\mathsf {pop}}\uplus \mathbb {T}_{\mathsf {noop}}\end{aligned}$$

As general notation, given a partial function *f*, we write $\mathsf {dom}(f),\mathsf {cod}(f)$ for the sets $\{i\ |\ f(i)\text { defined}\}$ and $\{j\ |\ \exists i.\,f(i)=j\}$ respectively. For each $1\le i<j\le n$, [*i*, *j*] is the set $\{i,i{+}1,\cdots ,j\}$.

#### Definition 21

We introduce the following notions.

- $\mathbb {L}=\mathbb {C}\cup \{\,{\texttt {R}}_{i}\ |\ 1\le i\le n\,\}$ is the set of *symbolic labels*. We use $\ell$ to range over its elements.
- $\mathsf {Reg}$ is the set of injective partial functions $\rho :\{1,\cdots ,n\}\rightharpoonup \mathbb {A}$. Its elements are called *register assignments* and we use $\rho$ to range over them.
- $\mathsf {Sto}$ is the set of partial functions $\varSigma {:}\mathbb {A}\rightharpoonup [0, max ]\cup \mathbb {A}$ such that $\mathsf {dom}{(\varSigma )}$ contains at most *n* elements and, moreover, if $\varSigma (a)=v$ then: if $a\in \mathbb {A}_{\mathsf {int}}$ then $v\in [0, max ]$; if $a\in \mathbb {A}_{\mathsf {ref}\, \gamma }$ then $v\in \mathbb {A}_{\gamma }\cap \mathsf {dom}(\varSigma )$ (i.e. $\varSigma$ is closed and well-typed). Its elements will be called *stores* and ranged over by $\varSigma$.
- $\mathsf {SSto}$ is the set of partial functions $S{:}[1,n]\rightharpoonup [0, max ]\cup \{{\texttt {R}}_{1},\cdots ,{\texttt {R}}_{n}\}$ such that $[1,n_{\mathsf {r}}]\subseteq \mathsf {dom}(S)$ and, for each $i\in \mathsf {dom}(S)$, $\mathsf {depth}(S,i)$ is well-defined (and finite). The *depth* and the *full value* of an index $i\in \mathsf {dom}(S)$ are given respectively by:
  $$\begin{aligned} \mathsf {depth}(S,i) = {\left\{ \begin{array}{ll} 1 \\ 1+\mathsf {depth}(S,j) \end{array}\right. }\quad S^*(i) = {\left\{ \begin{array}{ll} S(i) &{} \text { if }S(i)\in \{0,..., max \} \\ ({\texttt {R}}_{j},S^*(j)) &{}\text { if }S(i)={\texttt {R}}_{j} \end{array}\right. } \end{aligned}$$
  The elements of $\mathsf {SSto}$ will be called *symbolic stores* and ranged over by *S*.^3^ The depth restriction ensures that symbolic stores are closed and acyclic.
- $\mathsf {Sta}=(\mathbb {C}_{\mathsf {stack}}\times \mathsf {Reg})^*$ is the set of *stacks*. We shall range over stacks by $\sigma$, and over elements of a stack $\sigma$ by $(s,\rho )$.
- $\mathsf {Mix}$ is the set of partial injections $\pi :[n_{\mathsf {r}}{+}1,n]{\rightharpoonup }[n_{\mathsf {r}}{+}1,n]$.^4^ For each $\pi$, we write $\overline{\pi }$ for the extension of $\pi$ on [1, *n*]: $\overline{\pi }=\pi \cup \{(i,i)\ |\ i\in [1,n_{\mathsf {r}}]\}$.
- $\mathsf {TL}$ is the set of *transition labels*, taken from the set:
  $$\begin{aligned}&(\mathcal {P}([n_{\mathsf {r}}{+}1,n])\times \mathbb {L}\times \mathbb {T}_{\mathsf {push}}\times \mathbb {C}_{\mathsf {stack}}\times \mathsf {Mix}\times \mathsf {SSto}) \\ \cup&(\mathcal {P}([n_{\mathsf {r}}{+}1,n])\times \mathbb {L}\times \mathbb {T}_{\mathsf {pop}}\times \mathbb {C}_{\mathsf {stack}}\times \mathsf {Mix}\times \mathsf {SSto}) \\ \cup&(\mathcal {P}([n_{\mathsf {r}}{+}1,n])\times \mathbb {L}\times \mathbb {T}_{\mathsf {noop}}\times \mathsf {SSto}) \end{aligned}$$
  We range over $\mathsf {TL}$ by $\nu X.(\ell ,t,\phi )^S$, where $\phi$ can either be:
  - a push pair $(s,\pi )$, in which case we may also write $\nu X.(\ell ,t)^S/(s,\pi )$;
  - a pop pair $(s,\pi )$, in which case we may also write $\nu X.(\ell ,t)^S,(s,\pi )$;
  - or a no-op (), in which case we may simply write $\nu X.(\ell ,t)^S$.
  We stipulate that *S*(*j*) be defined whenever $\nu X.(\ell ,t,\phi )^S\in \mathsf {TL}$ and $j\in X$ or $\ell ={\texttt {R}}_{j}$. Moreover, we partition $\mathsf {TL}=\mathsf {TL}_{\mathsf {push}}\uplus \mathsf {TL}_{\mathsf {pop}}\uplus \mathsf {TL}_{\mathsf {noop}}$ depending on the partitioning of tags (e.g. $\mathsf {TL}_{\mathsf {push}}=\{\nu X.(\ell ,t,\phi )^S\ |\ t\in \mathbb {T}_{\mathsf {push}}\}$).

We write $\overline{\pi }(S)$ for $\{(\overline{\pi }(i),{\texttt {R}}_{\overline{\pi }(j)})\ |\ (i,{\texttt {R}}_{j})\in S\}\cup \{(\overline{\pi }(i),j)\ |\ (i,j)\in S\}$. Given a pair $(\rho ,S)\in \mathsf {Reg}\times \mathsf {SSto}$ we say that $\rho ,S$ are *compatible* if $\mathsf {dom}(S)=\mathsf {dom}(\rho )$ and, for all $i\in \mathsf {dom}(S)$,

$$\begin{aligned} \rho (i)\in \mathbb {A}_{\mathsf {int}}\implies S(i)\in \{0,\dots , max \}, \;\; \rho (i)\in \mathbb {A}_{\mathsf {ref}\, \gamma }\implies S(i)={\texttt {R}}_{j}\wedge \rho (j)\in \mathbb {A}_{\gamma }. \end{aligned}$$

In such a case, we can derive the store:

$$\begin{aligned} \mathsf {Sto}(\rho ,S) = \{\,(\rho (i),S(i))\ |\ S(i)\in \{0,..., max \}\,\} \cup \{\,(\rho (i),\rho (j))\ |\ S(i)={\texttt {R}}_{j}\,\} \end{aligned}$$

Moreover, we shall be using the following notation for assignment updates,

$$\begin{aligned} \rho [(i_1,...,i_m)\mapsto (z_1,...,z_m)] = \{(i,\rho (i))\ |\ i\in X\} \cup \{(i_j,z_j)\ |\ 1\le j\le m,z_j\not =\sharp \} \end{aligned}$$

with $X=\mathsf {dom}(\rho ){\setminus }{\{i_1,\cdots ,i_m\}}$ and each $z_j\in \mathbb {A}\cup \{\sharp \}$. Note in particular that the symbol $\sharp$ is used for register deletions. Similar notations will be used for store and partial-injection updates. Furthermore, for a store $\varSigma$ and a set of names *B*, we define $\varSigma \upharpoonright B=\{(a,v)\in \varSigma \ |\ a\in B\}$ and $\varSigma {\setminus } B=\{(a,v)\in \varSigma \ |\ a\notin B\}$. Finally, we let $\mathsf {clo}(\varSigma ,B)$ be the least set of names *C* such that $B\subseteq C$ and, for all $a\in C$, if $\varSigma (a)\in \mathbb {A}$ then $\varSigma (a)\in C$. For each symbolic store *S* and set of indices *X*, we define $S\upharpoonright X$, $S{\setminus } X$ and $\mathsf {clo}(S,X)$ in an analogous manner.

We can now define $(n_{\mathsf {r}},n)$-automata, which will be used for representing game semantics. An $(n_{\mathsf {r}},n)$-automaton is equipped with *n* registers, the first $n_{\mathsf {r}}$ of which will be read-only, and utilises a pushdown stack where it pushes stack symbols along with full register assignments.

#### Definition 22

An ($n_{\mathsf {r}},n$)-***automaton*** of type $\theta$ is given as a quintuple $\mathcal {A}=\langle Q,q_0,\rho _0,\delta ,F\rangle$ where:

- *Q* is a finite set of states, partitioned into $Q_{O}$ (*O*-states) and $Q_{P}$ (*P*-states);
- $q_0\in Q_{P}$ is the initial state; $F\subseteq Q_{O}$ is the set of final states;
- $\rho _0\in \mathsf {Reg}$ is the initial register assignment such that $[1,n_{\mathsf {r}}]\subseteq \mathsf {dom}(\rho _0)$;
- $\delta \subseteq (Q_P\times (\mathsf {TL}_{\mathsf {push}}\cup \mathsf {TL}_{\mathsf {noop}})\times Q_O) \cup (Q_O\times (\mathsf {TL}_{\mathsf {pop}}\cup \mathsf {TL}_{\mathsf {noop}})\times Q_P) \cup ( Q_O\times \mathsf {Mix}\times Q_O)\cup (Q_P\times \mathsf {Mix}\times Q_P)$ is the transition relation.

Additionally, if $\theta$ is a base type then there is a unique final state $q_F$ without outgoing transitions and reachable only via no-op transitions.

Our automata operate on words over the infinite alphabet $(\mathbb {C}\cup \mathbb {A})\times \mathbb {T}\times \mathsf {Sto}$. We shall write $(l,t)^\varSigma$ to refer to its elements. We first explain the meaning of the transition relation informally. Suppose $\mathcal {A}$ is at state $q_1$, $\rho$ is the current register assignment and $\sigma$ is the current stack.

- If $(q_1,\nu X.(\ell ,t,\phi )^S,q_2)\in \delta$, $\mathcal {A}$ will accept an input $(l,t)^\varSigma$ and move to state $q_2$ if the following steps are successful.
  - If $t\in \mathbb {T}_{\mathsf {pop}}$ and $\phi =(s,\pi )$, $\mathcal {A}$ will check whether the stack has the form $\sigma =(s,\rho ')::\sigma '$ with $\rho (i)=\rho '(i')\in \mathbb {A}$ iff $\pi (i)=i'$, for all $i,i'$, and $\mathsf {dom}(\rho )\cap \mathsf {dom}(\rho ')=\emptyset$. In such a case $\mathcal {A}$ will pop from the stack, that is, it will set $\sigma =\sigma '$ and $\rho =\rho [(i_1,...,i_m)\mapsto (\rho '(i_1),...,\rho '(n_m))]$, where $i_1,...,i_m$ is an enlisting of $\mathsf {dom}(\rho '){\setminus }\mathsf {cod}(\pi )$.
  - $\mathcal {A}$ will update $\rho$ with fresh names, that is, it will check whether $\mathsf {dom}(\rho )\cap X=\emptyset$ and, if so, it will set $\rho =\rho [(i_1,\cdots ,i_m)\mapsto (a_1,\cdots ,a_m)]$, where $i_1,\cdots ,i_m$ is an enumeration of *X* and $a_1,\cdots ,a_m$ are distinct names such that:
    - if $q_1\in Q_O$ then $a_1,\cdots ,a_m\notin \rho ([1,n])$ (*locally fresh*),
    - if $q_1\in Q_P$ then $a_1,\cdots ,a_m$ have not appeared in the current run of $\mathcal {A}$ (*globally fresh*).
  - $\mathcal {A}$ will check if $(l,\varSigma )$ corresponds to $(\ell ,S)$ via $\rho$, that is, whether $\varSigma =\mathsf {Sto}(\rho ,S)$ and either $\ell =l\in \mathbb {C}$, or $\ell ={\texttt {R}}_{i}$ and $\rho (i)=l$.
  - If $t\in \mathbb {T}_{\mathsf {push}}$ and $\phi =(s,\pi )$, $\mathcal {A}$ will perform a push of the registers in $\mathsf {dom}(\pi )$, after rearranging them according to $\pi$, that is, it will set $\sigma =(s,\rho \circ \pi )::\sigma$.
- If $(q_1,\pi ,q_2)\in \delta$, for $\pi \in \mathsf {Mix}$, $\mathcal {A}$ will reorganize the contents of registers in $[n_{\mathsf {r}}{+}1,n]$ according to $\pi$, that is, set $\rho =\rho \circ \overline{\pi }$, and move to $q_2$ without reading any input symbol ($\epsilon$-transition).

The above is formalized next. A *configuration* of $\mathcal {A}$ is a quadruple $(q,\rho ,\sigma ,H)\in {\hat{Q}}$, where ${\hat{Q}}=Q\times \mathsf {Reg}\times \mathsf {Sta}\times \mathcal {P}_{\!\mathsf {fn}}(\mathbb {A})$ and $\mathcal {P}_{\!\mathsf {fn}}(\mathbb {A})$ is the set of finite subsets of $\mathbb {A}$.

#### Definition 23

Let $\mathcal {A}=\langle Q,q_0,\rho _0,\delta ,F\rangle$ be an $(n_{\mathsf {r}},n)$-automaton. The configuration graph  of $\mathcal {A}$ is defined as follows (transitions are labelled by $\epsilon$ or elements of $(\mathbb {C}\cup \mathbb {A})\times \mathbb {T}\times \mathsf {Sto}$). For all $(q,\rho ,\sigma ,H)\in \hat{Q}$ and $(q,{\nu X.}(\ell ,t,\phi )^S,q')\in \delta$ we have  where $\varSigma =\mathsf {Sto}(\rho ',S)$ and:

- if $t\in \mathbb {T}_{\mathsf {pop}}$ and $\phi =(s,\pi )$ then $\sigma =(s,\rho _0)::\sigma '$ and
  - for all $i,i'$, $\rho (i)=\rho _0(i')$ iff $(i,i')\in \pi$, and $\mathsf {dom}(\rho _0)\cap \mathsf {dom}(\rho )=\emptyset$,
  - $$\rho _1=\rho [(i_1,...,i_m)\mapsto (\rho _0(i_1),...,\rho _0(i_m))]$$ with $$\{i_1,...,i_m\}=\mathsf {dom}(\rho _0){{\setminus }}\mathsf {cod}(\pi )$$;
  otherwise $$\rho _1=\rho $$;
- if $X=\{i_1,\cdots ,i_m\}$ then $\mathsf {dom}(\rho _1)\cap X=\emptyset$, $H_1=H\cup \{a_1,\cdots ,a_m\}$ and $\rho '=\rho _1[(i_1,\cdots ,i_m)\mapsto (a_1,\cdots ,a_m)]$ where $a_1,\cdots ,a_m$ are distinct names and:
  - if $$q\in Q_{O}$$ then $$a_1,\cdots ,a_m\notin \rho _1([1,n])$$,
  - if $$q\in Q_{P}$$ then $$a_1,\cdots ,a_m\notin \rho _1([1,n])\cup H$$;
- if $\ell \in \mathbb {C}$ then $l=\ell$ and $H'=H_1$;
- if $\ell ={\texttt {R}}_{i}$ then $l=\rho '(i)$ and $H'=H_1\cup \{l\}$;
- if $t\in \mathbb {T}_{\mathsf {push}}$ and $\phi =(s,\pi )$ then $\sigma '=(s,\rho '\circ \pi )::\sigma$.

Moreover, for all $(q,\rho ,\sigma ,H)\in \hat{Q}$ and $(q,\pi ,q')\in \delta$ we have , where $\rho '=\rho \circ \overline{\pi }$.

The set of strings *accepted* by $\mathcal {A}$ is defined as below, where $\epsilon$ is the empty stack.

.

We say that $\mathcal {A}$ is ***deterministic*** if, for any reachable configuration $\hat{q}$, any $x_1, x_2\in \{\epsilon \}\cup (\mathbb {C}\cup \mathbb {A})\times \mathbb {T}\times \mathsf {Sto}$, and any , if $x_1=x_2$ then $\hat{q}_1=\hat{q}_2$. The automata specifically used for our constructions follow some stronger disciplines.

#### Definition 24

We say that $\mathcal {A}$ is *strongly deterministic* if:

- for each $q\in Q_{P}$ there is at most one transition out of *q* (i.e. $|\delta \upharpoonright \{q\}|\le 1$), and if $(q,\nu X.(\ell ,t,\phi )^S,q')\in \delta$ then $|\delta \upharpoonright \{q'\}|\le 1$ and in particular $q'$ may only have an outgoing transition of the form $(q',\pi ,q'')$ such that $\forall \pi ',q'''.\ (q'',\pi ',q''')\notin \delta$;
- for each $q\in Q_{O}$ and $(q,\nu X_i.(\ell _i,t,\phi )^{S_i},q_i)\in \delta$, $i=1,2$, if $\nu X_1.(\ell _1,S_1)$ and $\nu X_2.(\ell _2,S_2)$ are equal up to permutation of indices^5^ in $X_1,X_2$ then $\nu X_1.(\ell _1,t,\phi )^{S_1} = \nu X_2.(\ell _2,t,\phi )^{S_2}$ and $q_1=q_2$;
- for each $(q,\nu X.(\ell ,t,\phi )^S,q')\in \delta$, *X* is contained in $\mathsf {clo}(S,X_{\mathsf {Av}})$ where $X_{\mathsf {Av}}=(\mathsf {dom}(S){\setminus } X)\cup \{j\ |\ \ell ={\texttt {R}}_{j}\}$.

For such an $\mathcal {A}$, we may write $q_P\xrightarrow {\nu X.(\ell ,t,\phi )^S;\pi }q_O$ for $q_P\xrightarrow {\nu X.(\ell ,t,\phi )^S}q_O'\xrightarrow {\pi }q_O$, where $q_P\in Q_{P}$, $q_O\in Q_{O}$. The last condition above corresponds to *frugality* (cf. Definition 5): fresh names must be reachable from names that were already available or appear in the current transition label.

#### Lemma 25

If $\mathcal {A}$ is strongly deterministic then it is deterministic.

#### Proof

The claim is obvious for configurations with P-states, as well as for (reachable) configurations with O-states and outgoing transitions of the form $q\xrightarrow {\pi }q'$, because of the first condition in the previous definition. For configurations with O-states and transitions of the form $q\xrightarrow {\nu X.(\ell ,t,\phi )^S}q'$, the second condition above ensures that each label has at most one accepting edge, as long as in each configuration each top stack element can be popped uniquely (i.e. with at most one $\phi$). The latter follows from the definition of configuration graphs. $\square$

#### Lemma 26

Given $\mathcal {A}$ strongly deterministic and $w_1,w_2\in L(\mathcal {A})$, if $\mathrm {ext}(w_1)\cap \mathrm {ext}(w_2)\not =\emptyset$ then $w_1=w_2$.

#### Proof

WLOG we assume that $w_1,w_2$ have the same underlying sequence of moves. Let $w_i'm^{\varSigma _i}$ be the prefix of $w_i$ of length *n* ($i=1,2$). We show by induction on *n* that $\varSigma _1=\varSigma _2$. By IH (if $n>1$) or by definition (if $n=1$) we have that $w_1'=w_2'$. Moreover, by the previous lemma, $w_1',w_2'$ lead to some common configuration $\hat{q}$ of $\mathcal {A}$. If *n* is even then, by the first clause of Definition 24, we have that $\varSigma _1=\varSigma _2$. If *n* is odd then by hypothesis we have that $\varSigma _1,\varSigma _2$ have a common extension and, thus, using the last clause of Definition 24, we obtain $\varSigma _1=\varSigma _2$. $\square$

#### Definition 27

Let $\mathcal {A}=\langle \, Q,q_0,\rho _0,\delta ,q_F \,\rangle$ be a strongly deterministic automaton of base type. We define the set of *quasi-final* states *E* to be the set of states that reach $q_F$ in one step. Then *E* is canonically partitioned as $E = \biguplus \nolimits _{(X,\ell ,t,S)}E_{\nu X.(\ell ,t)^S}$ where $E_{\nu X.(\ell ,t)^S}=\{\,q\in Q\ |\ (q,\nu X.(\ell ,t,())^S,q_F)\in \delta \,\}$ and $\mathcal {A}$ is uniquely determined by the structure $\mathcal {A}^{-}=\langle Q,q_0,\rho _0,\delta ,E\rangle$.

### Automata for 

Recall we are only going to translate terms in canonical form.

Let $\varGamma =\{x_1:\theta _1,\cdots ,x_m:\theta _m\}$ and ${\varGamma } \vdash {\mathbb {C}:\theta }$ be a -term in canonical form. Let us write $P_{{\varGamma } \vdash {\theta }}^1$ for the set of plays-with-store of length 1 over ${\varGamma } \vdash {\theta }$. Recall that each of them will have the form $\mathrm {i}^{\varSigma _0}$, where $\mathrm {i}\in I_{\varGamma }$, i.e. $\mathrm {i}=(l_1,\cdots ,l_m)$ with $l_i\in \mathcal {L}_{\theta _i}$. Moreover, the names in $\mathrm {i}^{\varSigma _0}$ coincide with those of $\mathsf {dom}(\varSigma _0)=\nu (\varSigma _0)$ and, by frugality, $\nu (\varSigma _0)=\mathsf {clo}(\varSigma _0,\nu (\mathrm {i}))$. These can be ordered by use of register assignments, of fixed size appropriate to contain all names in $\varSigma _0$ and names created while translating -terms, leading to the following construction.

$$\begin{aligned} I_{{\varGamma } \vdash {\theta }}^+= \{(\mathrm {i}^{\varSigma _0},\rho _0)\ |\ \mathrm {i}^{\varSigma _0}\in P_{{\varGamma } \vdash {\theta }}^1,\,\nu (\rho _0)=\nu (\varSigma _0),\,\exists k.\,\rho _0([1,k])=\nu (\mathrm {i})\} \end{aligned}$$

For brevity, we shall write each element $(\mathrm {i}^{\varSigma _0},\rho _0)\in I_{{\varGamma } \vdash {\theta }}^+$ as $\mathrm {i}^{\varSigma _0}_{\rho _0}$. We now instantiate the automata defined in the previous section by using the finite set of tags $\mathbb {T}=\mathbb {T}_{\mathsf {push}}\cup \mathbb {T}_{\mathsf {pop}}\cup \mathbb {T}_{\mathsf {noop}}$, where

$$\begin{aligned} \mathbb {T}_{\mathsf {push}}&= \{\mathsf {c}_i\in \mathbb {T}\ |\ i>0\}\cup \{\mathsf {c}_i^x\in \mathbb {T}\}\cup \{\mathsf {c}_{j,i}^x\in \mathbb {T}\ |\ i>0\}\\ \mathbb {T}_{\mathsf {pop}}&= \{\mathsf {r}_i\in \mathbb {T}\ |\ i>0\}\cup \{\mathsf {r}_i^x\in \mathbb {T}\}\cup \{\mathsf {r}_{j,i}^x\in \mathbb {T}\ |\ i>0\}\\ \mathbb {T}_{\mathsf {noop}}&={\{\mathsf {r}_{\downarrow }, \mathsf {c}_0, \mathsf {r}_0, \mathsf {c}_{j,0}^x, \mathsf {r}_{j,0}^x\}}. \end{aligned}$$

Moreover, we will impose the following condition on our automata. All push/pops will not involve any registers (i.e. they will have $\phi =(s,\emptyset )$), except if the tag *t* in question satisfies:

$$\begin{aligned} t\in \{\mathsf {c}_i,\mathsf {r}_i\in \mathbb {T}\ |\ i>0\}\cup \{\mathsf {c}_{j,i}^x,\mathsf {r}^x_{j,i}\in \mathbb {T}\ |\ i>0\} \end{aligned}$$

#### Remark 28

A canonical form of  will be translated into a family of automata indexed by $I_{{\varGamma } \vdash {\theta }}^+$. For each $\mathrm {i}^{\varSigma _0}_{\rho _0}\in I_{{\varGamma } \vdash {\theta }}^+$, the corresponding automaton will accept exactly the words *w* such that $\mathrm {i}^{\varSigma _0} w$ is a representation of a complete play induced by the canonical form. The family will be infinite, but *finite* when considered up to name permutations.

For any -term $\varGamma \vdash \mathbb {C}:\theta$ in canonical form we define an $I_{{\varGamma } \vdash {\theta }}^+$-indexed family of automata  (each of type $\theta$) by induction on the shape of $\mathbb {C}$. In all cases  will have $n_0=|\nu (\mathrm {i})|$ read-only registers and the initial assignment will be $\rho _0$. The precise number of registers can be calculated easily by reference to the constituent automata. Let us write $S_0$ for the symbolic store defined by $S_0(i)=\varSigma _0(\rho _0(i))$ if $\varSigma _0(\rho _0(i))\in \{0,\dots , max \}$, and $S_0(i)={\texttt {R}}_{j}$ if $\varSigma _0(\rho _0(i))=\rho _0(j)$. The base and inductive cases are as follows.

.
.
, where $$x\equiv x_k$$ and $$l_k=\rho _0(j)$$.
, where $$x\equiv x_k$$ and $$l_k=j.$$
where $x\equiv x_k$, $y\equiv x_j$ and $\varSigma '_0=\varSigma _0[l_k\mapsto l_j]$, and the initial transition deletes all names in $\rho _0$ which break frugality of $\mathrm {i}^{\varSigma _0'}$, that is, $\pi (i)=i$ just if $\rho _0(i)\in \mathsf {clo}(\varSigma _0',\nu (\mathrm {i}))$. Moreover, $\rho _0'=\rho _0\circ \overline{\pi }$ and $\varSigma _0''=\varSigma _0'\upharpoonright \mathsf {cod}(\rho _0').$
where $x\equiv x_k$ and $\pi$ transfers $\varSigma _0(l_k)$, if it is a name, to the register in position $n_0+1$, and leaves all other names in $\rho _0$ untouched. Moreover, $\rho _0'=\rho _0\circ \overline{\pi }$.
where *z* has arity *r* and $\varSigma$ ranges over stores with $\mathsf {dom}(\varSigma )=\mathsf {clo}(\varSigma ,\nu (\mathrm {i}))$. These are infinitely many, but finitely many up to permutations of fresh names. In the transitions we pick *X*, *S* such that there is one transition for each of the (finitely many) equivalence classes. Moreover, $\rho _0'$ is specified by stipulating $\mathsf {dom}(\rho _0')=[1,n_0]\cup X$ and $\varSigma =\mathsf {Sto}(\rho _0',S)$.
with $r,\varSigma ,S,X,\rho _0'$ as above and *j* ranging over $[0, max ]$.
where $r,\varSigma ,S,X,\rho _0'$ are as above, *j* ranges over elements of $\mathsf {dom}(S)$ such that $\mathsf {ref}\, \gamma =\mathsf {ref}^{\mathsf {depth}(S,j)}\, \mathsf {int}$, and $b\in \mathbb {A}_{\gamma }$ is a fresh name. We let $\varSigma '$ range over all stores with $\mathsf {dom}(\varSigma ')=\mathsf {clo}(\varSigma ',\nu (\mathrm {i})\cup \{b\})$. We pick $X',S'$ such that there is one symbolic transition for each of the intended transitions $(b,\mathsf {r}_r^z)^{\varSigma '}$ and specify $\rho _0''$ accordingly, making sure that $\rho _0''(n_0+1)=b$.
where $r,\varSigma ,S,X,\rho _0'$ are as above and  is  where we have replaced every tag superscript *y* with *z*.
and  are defined similarly to the above.
- Case of $\mathsf {let}\,x^{\mathsf {ref}^2\, \gamma }=\mathsf {ref}(y^{\mathsf {ref}\, \gamma })\,\mathsf {in}\,\mathbb {C}$. Here the inductive hypothesis gives us an automaton for $\varGamma ,x\,{:}\,\mathsf {ref}^2\, \gamma \vdash \mathbb {C}\,{:}\,\theta$. In order to transform the latter into an automaton for our given term, we need to hide the name corresponding to *x* from the automaton, until the point where the name is eventually revealed in some move (it is also possible that the name remains private indefinitely). This hiding of *x* effectively has wider repercussions as we need also to hide any name that *x* exclusively points to, and so on, along with their stored values. It is therefore useful to define a more general hiding construction.

Let $\mathcal {A}$ be an $(n_{\mathsf {r}},n)$-automaton, let $X_0,\cdots ,X_h$ be an enumeration of all subsets of $[n_{\mathsf {r}}+1,n]$ and let $T_{i,0},\cdots ,T_{i,g_i}$ be an enumeration of all partial symbolic stores on $X_i$ (i.e. of all $T=S\upharpoonright X_i$ for some symbolic store *S*). For each $0\le i\le h$ and $0\le j\le g_i$ we define an $(n_{\mathsf {r}},n)$-automaton $\mathcal {A}_{X_i}^{T_{i,j}}$ to be a copy of $\mathcal {A}$ in which we have hidden the names in registers $X_i$, while the stored restricted to the names in those registers is $T_{i,j}$. Concretely, $\mathcal {A}_{X_i}^{T_{i,j}}$ is a copy of $\mathcal {A}$ in which we have removed all transitions apart from those of the form $q_1\xrightarrow {\nu X.(\ell ,t,\phi )^S}q_2$ with $q_1$ an O-state and such that:

$$\begin{aligned} S\upharpoonright X_i=T_{i,j}\,,\; \forall m\in X_i.\forall m'\notin X_i.\ \ell \not ={\texttt {R}}_{m},\ S(m')\not ={\texttt {R}}_{m} \end{aligned}$$

and in those remaining transitions we have removed $X_i$ from the domains of all symbolic stores. We define $\nu \mathcal {A}$ as the $(n_{\mathsf {r}},n)$-automaton fragment (no initial state) obtained by interconnecting these automata as below.The transitions are as follows. Let (*X*, *T*) be an element of the above enumeration.

- For each $q_1\xrightarrow {\nu Y.(\ell ,t,\phi )^S}q_2$ in $\mathcal {A}$ with $q_1$ a *P*-state, add a transition from $q_1$ in $\mathcal {A}_X^T$ to $q_2$ in $\mathcal {A}_{X'}^{T'}$ with label $\nu Y'.(\ell ,t,\phi )^{S'}$, where $X'=(X\cup Y){\setminus } Y'$, $S'=S{\setminus } X'$, $T'=S\upharpoonright X'$ and $Y' = \mathsf {clo}(S,(\mathsf {dom}(S){\setminus } X)\cup \{j\ |\ \ell ={\texttt {R}}_{j}\})$.
- For each $q_1\xrightarrow {\pi }q_2$ in $\mathcal {A}$ add a transition from $q_1$ in $\mathcal {A}_X^T$ to $q_2$ in $\mathcal {A}_{X'}^{T'}$ with label $\pi$, where $X'=\pi ^{-1}(X)$ and $T'=\pi ^{-1}(T)$.

Let now $a\in \mathbb {A}_{\mathsf {ref}\, \gamma }{\setminus }\nu (\rho _0)$, suppose $y\equiv x_k$, $\rho _0(k')=l_k$, let $\varSigma _0'=\varSigma _0[a\mapsto l_k]$ and let $n_0'$ be the first empty register in $\rho _0$. We take $\pi _0\in \mathsf {Mix}$ to be such that it swaps $n_0+1$ and $n_0'$, and fixes all other indices, and set $\rho _0'=\rho _0[n_0+1\mapsto a,n_0'\mapsto \rho _0(n_0+1)]$. We define:  where the transition $\pi _0$ points to the initial state of the $(\{n_0+1\},\{(n_0+1,{\texttt {R}}_{k'})\})$ component of .^6^

- The case of $\mathsf {let}\,x^{\mathsf {ref}\, \mathsf {int}}=\mathsf {ref}(0)\,\mathsf {in}\,\mathbb {C}$ is dealt with similarly to the above.
- For $(\mathsf {while}\,(!x)\,\mathsf {do}\,\mathbb {C});\mathbb {C}'$, given the automata  and , with appropriate initialisations, we construct a new automaton as follows. Suppose $x\equiv x_k$ and $\rho _0(k')=l_k$. If the initial value stored for *x* is 0 (i.e. if $\varSigma _0(l_k)=0$) then we simply return . Otherwise, we need to combine the automata for $\mathbb {C}$ and $\mathbb {C}'$ in such a away so that  is involved repeatedly (with appropriate initialisation), until it reaches a final state with a final transition with a symbolic store assigning 0 to $k'$. At this point, the automaton would switch and start simulating . An important point in this construction is that the final transitions of  are hidden in the new automaton, as the return values of the while guard are not revealed in the semantics. This hiding implies a potential hiding of names as well: any names created in final transitions of  need to be hidden as well. This latter kind of hiding is delegated to the $\nu$-construction that we described two cases above.

Formally, let $\varSigma _0,\cdots ,\varSigma _h$ be an enumeration (modulo permutation of fresh names) of all stores $\varSigma$ with $\mathsf {dom}(\varSigma )=\mathsf {clo}(\varSigma ,\nu (\mathrm {i}))$. Recall $x\equiv x_k$, $\rho _0(k')=l_k$, and recall the presentation of an automaton given in Definition 27. We define  to be  if $\varSigma _0(l_k)=0$. Otherwise, we define it to be a combination of ,  and , , with each $\rho _i$ specified by $\varSigma _i$ as above, connected together as below.The initial state is the one of the $(\emptyset ,\emptyset )$ component of . Let $S_i$ be specified by each $\varSigma _i,\rho _i$. For each quasi-final state $q\in E_{\nu X.(\star ,\mathsf {r}_\downarrow )^S}$ of each , there are unique *m* and $\pi$ such that $S^*(i)=(\overline{\pi }(S_m))^*(i)$ for all $i\in [1,n_0]$. Let $X'=\pi ^{-1}(X)$ and $T'=S_m\upharpoonright X'$. We add a transition labelled with $\pi$:

- if $S(k')=0$, from *q* to the initial state of the $(X',T')$ component of ,
- if $S(k')\ne 0$, from *q* to the initial state of the $(X',T')$ component of .

- Case of $\lambda x^{\mathsf {unit}\rightarrow \varTheta _1}.\mathbb {C}$. We define  as an automaton which combines states $q_0,q_1,q_2$ and two modified copies of , for each (of the finitely many relevant) $\varSigma$, in each of which we have replaced tags $\mathsf {r}_\downarrow$ by $\mathsf {r}_0$ and removed all transitions with tags $\mathsf {c}_i^x,\mathsf {r}_i^x$. We let *X*, *S* be derived from $\varSigma$ and denote the two copies by . Each state *q* in  has copies $\hat{q},\tilde{q}$ in  respectively.

The unique final state is $q_1$. The transitions in typewriter font are defined as follows.

- We connect every final state of  with $q_1$ using a transition with label $\emptyset$ ($\texttt {done}$). Similarly for $\texttt {done}'$.
- For each sequence $q_A\xrightarrow {\nu X_A.(\ell _A,\mathsf {c}^x_i)^{S_A}/(s,\emptyset )\,;\,\pi }q_B\xrightarrow {\nu X_B.(\ell _B,\mathsf {r}^x_i)^{S_B},(s,\emptyset )} q_C$ in  we add $\hat{q}_A\xrightarrow {\nu X_A.(\ell _A,\mathsf {c}_i)^{S_A}/(\hat{q}_A,\pi )\,;\,\emptyset } q_2\xrightarrow {\nu X_B.(\ell _B,\mathsf {r}_i)^{S_B},(\hat{q}_A,\emptyset )} \hat{q}_C$ ($\texttt {push}$, $\texttt {pop}$) and $\tilde{q}_A\xrightarrow {\nu X_A.(\ell _A,\mathsf {c}_i)^{S_A}/(\tilde{q}_A,\pi )\,;\,\emptyset }q_2\xrightarrow {\nu X_B.(\ell _B,\mathsf {r}_i)^{S_B},(\tilde{q}_A,\emptyset )}\tilde{q}_C$ ($\texttt {push}'$, $\texttt {pop}'$).

The other cases of $\lambda x^{\beta \rightarrow \varTheta _1}.\mathbb {C}$ are dealt with in a similar way. The case of $\lambda x^{\beta }.\mathbb {C}$ is treated as above, excluding $q_2$ and  from the construction.

- For the case of $\mathsf {let}\,y=z(\lambda x^{\mathsf {unit}\rightarrow \varTheta _1}.\mathbb {C})\,\mathsf {in}\,\mathbb {C}'$ it is useful to introduce a notion of automaton which operates by interleaving runs from two constituent automata. Since a similar construction will be of use in the next section, we give a general notion of automaton which can combine runs either by matching or by interleaving them. We define these generalised automata and give the construction of the one corresponding to $\mathsf {let}\,y=z(\lambda x^{\mathsf {unit}\rightarrow \varTheta _1}.\mathbb {C})\,\mathsf {in}\,\mathbb {C}'$. Generalised automata can be reduced to equivalent ones.

Let $n_{\mathsf {r}}\le n_1,n_2$ and set $n'=n_1+n_2-n_{\mathsf {r}}$. For each $i\in [n_{\mathsf {r}}+1,n_2]$ we define its *shift* $i^+=i+n_1-n_{\mathsf {r}}$; note that $i^+\in [n_1+1,n']$. We also fix a fresh label symbol $\checkmark$, which is used to indicate the automaton that will not advance in a given transition. For any set *X*, we write $X^\checkmark$ for $(X\uplus \{\checkmark \})^2{\setminus }\{(\checkmark ,\checkmark )\}$.

A *generalised* $(n_{\mathsf {r}},n_1,n_2)$-*automaton* is given as a quintuple $\mathcal {A}=\langle Q,q_0,\rho _0,\delta ,F\rangle$, where these components are defined as for an $(n_{\mathsf {r}},n')$-automaton except that now:

- $$\delta \subseteq (Q_P\times (\mathsf {TL}_{\mathsf {push}}\cup \mathsf {TL}_{\mathsf {noop}})^\checkmark \times Q_O) \cup (Q_O\times (\mathsf {TL}_{\mathsf {pop}}\cup \mathsf {TL}_{\mathsf {noop}})^\checkmark \times Q_P) \cup ( Q_O\times \mathsf {Mix}^\checkmark \times Q_O)\cup (Q_P\times \mathsf {Mix}^\checkmark \times Q_P)$$,
- $\rho _0\in \mathsf {Reg2}$ where $\mathsf {Reg2}$ contains all $\rho :[1,n']\rightharpoonup \mathbb {A}$ such that $\rho \upharpoonright [1,n_1]$ and $\rho \upharpoonright ([1,n_{\mathsf {r}}]\cup [n_1{+}1,n'])$ are both injective.

Moreover, apart from the usual partitioning to O- and P-states, *Q* is partitioned to *normal* and *divergent* states: $Q=Q_N\uplus Q_D$. There is a map $\mathsf {div}:Q_N\cap Q_{P}\rightarrow Q_D\cap Q_{P}$, while $q_0\in Q_N$.

The automaton operates on words over the alphabet $(\mathbb {C}\cup \mathbb {A})\times \mathbb {T}\times \mathsf {Sto}$, with configurations given by tuples $(q,\rho ,\sigma ,H,\varSigma )$, where now $\rho \in \mathsf {Reg2}$, $\varSigma \in \mathsf {Sto}$ and the stack $\sigma$ is an element of $\mathsf {Sta2}=(\mathbb {C}_{\mathsf {stack}}^\checkmark \times \mathsf {Reg2})^*$. We define projections and pairings for moving from the generalised setting to the ordinary one and vice versa:

- for each $\rho \in \mathsf {Reg2}$ let $\pi _1(\rho )=\rho \upharpoonright [1,n_1]$ and $\pi _2(\rho )=\rho \upharpoonright [1,n_{\mathsf {r}}]\cup \{(i,\rho (i^+))\ |\ i\in [n_{\mathsf {r}}{+}1,n_2]\}$;   moreover, for each $\rho _1,\rho _2\in \mathsf {Reg}$ let $\langle \rho _1,\rho _2\rangle =\rho _1\cup \{(i^+,\rho _2(i))\ |\ i\in [n_{\mathsf {r}}+1,n_2]\}$;
- for each $(s_1,s_2,\rho )::\sigma \in \mathsf {Sta2}$ we set $\pi _i((s_1,s_2,\rho )::\sigma )=(s_i,\pi _i(\rho ))::\pi _i(\sigma )$, and $\pi _i(\epsilon )=\epsilon$, for $i=1,2$; moreover, let $\langle (s_1,\rho _1)::\sigma _1,(s_2,\rho _2)::\sigma _2\rangle =(s_1,s_2,\langle \rho _1,\rho _2\rangle )::\langle \sigma _1,\sigma _2\rangle$ and $\langle \epsilon ,\epsilon \rangle =\epsilon$.

The automaton induces the following configuration graph. The initial configuration is $(q_0,\rho _0,\epsilon ,\emptyset ,\emptyset )$. For each $(q,\rho ,\sigma ,H,\varSigma )$ and $(q,\nu X_1.(\ell _1,t_1,\phi _1)^{S_1},\nu X_2.(\ell _2,t_2,\phi _2)^{S_2},q')\in \delta$ if $q\xrightarrow {\nu X_i.(\ell _i,t_i,\phi _i)^{S_i}}q'$ induces $(q,\pi _i(\rho ),\pi _i(\sigma ),H)\xrightarrow {(l_i,t_i)^{\varSigma _i}}(q',\rho _i,\sigma _i,H_i)$ on an ordinary $(n_{\mathsf {r}},n_i)$-automaton where

1. $$(l_1,t_1)=(l_2,t_2)$$,
2. if $q\in Q_{P}$ then $\varSigma [\varSigma _1]\cup \varSigma [\varSigma _2]$ is well-defined,^7^
3. if $q\in Q_{O}$ then $\varSigma _1\cup \varSigma _2$ is well-defined,

then $(q,\rho ,\sigma ,H,\varSigma )\xrightarrow {(l_1,t_1)^{\varSigma _1\cup \varSigma _2}}(q',\langle \rho _1,\rho _2\rangle ,\langle \sigma _1,\sigma _2\rangle ,H_1\cup H_2,\varSigma _1\cup \varSigma _2)$. Moreover, in case $q\in Q_{P}\cap Q_N$ and conditions 1,2 above cannot be satisfied by any combination of $l_i,t_i,\varSigma _i$ then the automaton *diverges*, that is, $(q,\rho ,\sigma ,H,\varSigma )\xrightarrow {\epsilon }(\mathsf {div}(q),\rho ,\sigma ,H,\varSigma )$.

If $(q,\nu X_1.(\ell _1,t_1,\phi _1)^{S_1},\checkmark ,q')\in \delta$ with $(q,\pi _1(\rho ),\pi _1(\sigma ),H)\xrightarrow {(l_1,t_1)^{\varSigma _1}}(q',\rho _1,\sigma _1,H_1)$ in an $(n_{\mathsf {r}},n_1)$-automaton, we have $(q,\rho ,\sigma ,H,\varSigma )\xrightarrow {(l_1,t_1)^{\varSigma '}}(q',\langle \rho _1,\pi _2(\rho )\rangle ,\langle \sigma _1,\sigma _2\rangle ,H_1,\varSigma ')$ where $\varSigma '=\varSigma [\varSigma _1]\upharpoonright \mathsf {cod}(\langle \rho _1,\pi _2(\rho )\rangle )$ and:

$$\begin{aligned} \sigma _2 = {\left\{ \begin{array}{ll} (\checkmark ,\emptyset )::\pi _2(\sigma ) &{} \text { if }t_1\in \mathbb {T}_{\mathsf {push}}\\ \sigma ' &{} \text { if }t_1\in \mathbb {T}_{\mathsf {pop}}\text { and }\pi _2(\sigma )=(s_2,\rho _2')::\sigma ' \\ \pi _2(\sigma ) &{} \text { if }t_1\in \mathbb {T}_{\mathsf {noop}}\\ \end{array}\right. } \end{aligned}$$

For each $(q,\pi _1',\pi _2',q')\in \delta$ we have $(q,\rho ,\sigma ,H,\varSigma )\xrightarrow {\epsilon }(q',\langle \rho _1,\rho _2\rangle ,\sigma ,H,\varSigma ')$ with $\rho _i=\pi _i(\rho )\circ \overline{\pi _i'}$ and $\varSigma '=\varSigma \upharpoonright \mathsf {cod}(\langle \rho _1,\rho _2\rangle )$. If $(q,\pi _1',\checkmark ,q')\in \delta$ then $(q,\rho ,\sigma ,H,\varSigma )\xrightarrow {\epsilon }(q',\langle \rho _1,\pi _2(\rho )\rangle ,\sigma ,H,\varSigma \upharpoonright \mathsf {cod}(\langle \rho _1,\pi _2(\rho )\rangle ))$. For each $(q,\checkmark ,z,q')\in \delta$ we do the analogous of the symmetric case above.

Note in particular that if $\mathcal {A}$ only contains transitions which include $\checkmark$ then we can drop the component $\varSigma$ in configurations and disregard divergence. In fact, any $(n_{\mathsf {r}},n_1)$-automaton can be rendered into an $(n_{\mathsf {r}},n_1,n_2)$-automaton by simply changing each transition $(q,z,q')$ to $(q,z,\checkmark ,q')$. The dual applies to every $(n_{\mathsf {r}},n_2)$-automaton.

We now proceed with the case of $\mathsf {let}\,y=z(\lambda x^{\mathsf {unit}\rightarrow \varTheta _1}.\mathbb {C})\,\mathsf {in}\,\mathbb {C}'$. The corresponding automaton will first read a move indicating that *z* is being called (tag $\mathsf {c}_r^z$). After that, a detour to $\lambda x.\mathbb {C}$ will be an option, which O can initiate with a move tagged with $\mathsf {c}_r^{z,0}$. Modelling the detour is analogous to the interpretation of $\lambda x.\mathbb {C}$. Once the detour is completed or the possibility is not exercised, O can play a move tagged with $\mathsf {r}_r^z$ corresponding to the return by *z* (tagged $\mathsf {r}_r^z$). This will trigger a transition to the automaton for $\mathbb {C}'$, in which we need to modify labels by replacing *y* with *z* and to allow for detours to $\lambda x.\mathbb {C}$ each time $\mathbb {C}'$ makes a transition on a P-move corresponding to *y*. Note that this is consistent with the behaviour of the corresponding strategy, due to the visibility and well-bracketing conditions.

Technically, for each $\varSigma '$ and related $S',X',\rho _0''$, we consider two modified copies of  in which:

- all O-states *q* for which there are $q_A\xrightarrow {\nu X_A.(\ell _A,t_A,\phi _A)^{S_A}\,;\,\pi } q\xrightarrow {\nu X_B.(\ell _B,t_B,\phi _B)^{S_B}}q_B$ in ,
  with $t_A$ having superscript *y*, are tagged as $q_{\mathbb {C}'}$;
- we replace tag superscripts *y* with *z*.

We set $n_2$ to be the maximum number of registers in these automata, and let $n=n_1+n_2-n_0$ with $n_1$ defined below. We denote the two copies by  and  respectively and consider them to be $(n_0,n_1,n_2)$-automata. In the first one we write states as $\hat{q}$, while the other one has (the same) states in form $\tilde{q}$.

For each tagged state $\hat{q}_{\mathbb {C}'}$ of , and each $\varSigma$ and related $X,S,\rho _0'$, consider the automaton . We set $n_1$ to be the maximum number of registers in these automata. We define a modified copy of each  by:

- removing all transitions of the form $q_A\xrightarrow {\nu X_A.(\ell _A,\mathsf {c}_i^x,\phi _A)^{S_A};\,\pi \!\!\!}q_B\xrightarrow {\nu X_B.(\ell _B,\mathsf {r}_i^x,\phi _B)^{S_B}\!\!\!\!\!} q_C$;
- replacing all tags $\mathsf {r}_\downarrow$ by $\mathsf {r}^z_{r,0}$, where *r* is the arity of *z*.

For each *q* in  we denote the resulting automaton by , considered as an $(n_0,n_1,n_2)$-automaton, and its states by $(q,\hat{q}_{\mathbb {C}'},S)$. We construct another copy  following the same routine, albeit for each state $\tilde{q}_{\mathbb {C}'}$ of .

We construct an $(n_0,n_1,n_2)$-automaton for  as follows.All the arrows above represent transitions in the first partition of the automaton (but we have omitted the RHS $\checkmark$ for economy), and the same goes for all transitions inside subautomata involving $\mathbb {C}$. The transitions in subautomata coming from $\mathbb {C}'$ contribute to the second component. We connect each state $\hat{q}_{\mathbb {C}'}$ of  with the initial state of  (for each relevant $S,S'$), using a transition with label $\nu X.(\star ,\mathsf {c}_{r,0}^z)^{S}$. Similarly for the transition of the same label between  and . The transitions in typewriter font in the first line of the diagram are as in the previous case. Those of the second line are explained below.

- We connect every final state of each  with $\hat{q}_{\mathbb {C}'}$ using a transition with label $\emptyset$ ($\texttt {done}$). Similarly for $\texttt {done}'$.
- For each $q_{\mathbb {C}'},S$ and each $q_A\xrightarrow {\nu X_A.(\ell _A,\mathsf {c}_i^x)^{S_A}/(s,\emptyset )\,;\,\pi }q_B\xrightarrow {\nu X_B.(\ell _B,\mathsf {r}_i^x)^{S_B},(s,\emptyset )} q_C$ in  we add ($\texttt {push}$, $\texttt {pop}$)
  $$\begin{aligned} (q_A,\hat{q}_{\mathbb {C}'},S)\xrightarrow {\nu X_A.(\ell _A,\mathsf {c}_{r,i}^z)^{S_A}/{\phi }_A\,;\,\emptyset } \hat{q}_{\mathbb {C}'}\xrightarrow {\nu X_B.(\ell _B,\mathsf {r}_{r,i}^z)^{S_B},{\phi }_B} (q_C,\hat{q}_{\mathbb {C}'},S) \end{aligned}$$
  with ${\phi }_A=((q_A,\hat{q}_{\mathbb {C}'},S),\pi )$ and ${\phi }_B=((q_B,\hat{q}_{\mathbb {C}'},S),\emptyset )$. We also add ($\texttt {push}'$, $\texttt {pop}'$) $(q_A,\tilde{q}_{\mathbb {C}'},S)\xrightarrow {\nu X_A.(\ell _A,\mathsf {c}_{r,i}^z)^{S_A}/{\phi }_A\,;\,\emptyset } \tilde{q}_{\mathbb {C}'}\xrightarrow {\nu X_B(\ell _B,\mathsf {r}_{r,i}^z)^{S_B},{\phi }_B} (q_C,\tilde{q}_{\mathbb {C}'},S)$ setting ${\phi }_A=((q_A,\tilde{q}_{\mathbb {C}'},S),\pi )$ and ${\phi }_B=((q_B,\tilde{q}_{\mathbb {C}'},S),\emptyset )$.

The other cases of $\mathsf {let}\,y=z(\lambda x^{\beta \rightarrow \varTheta _1}.\mathbb {C})\,\mathsf {in}\,\mathbb {C}'$ are dealt with in a similar way.

Observe that the basic cases in our construction yield strongly deterministic automata. Moreover, strong determinacy is preserved in the inductive cases. This is obvious in cases without interaction between different sub-automata. Moreover, in the cases of $\mathsf {let}\,x=\mathsf {ref}(y)\,\mathsf {in}\,\mathbb {C}$ and $(\mathsf {while}\,(!x)\,\mathsf {do}\,\mathbb {C});\mathbb {C}'$, the connections between different components are made from states which end up having unique outgoing transitions. The same holds also for the cases of $\lambda x.\mathbb {C}$ and $\mathsf {let}\,y=z(\lambda x.\mathbb {C})\,\mathsf {in}\,\mathbb {C}'$, with the addition that now there are also new pop transitions to be taken into account which, however, operate on fresh stack symbols and therefore do not interfere with the constituent automata. In the latter case, the reduction from the generalised interleaving automaton to the ordinary one preserves strong determinacy.

Recall from Remark 28 that  and $\llbracket \cdots \rrbracket$ merely differ by the absence of initial moves in . Consequently, the constructions outlined above imply the following lemma.

#### Lemma 29

Let ${\varGamma } \vdash {\mathbb {C}:\theta }$ be a -term in canonical form. For each $j=\mathrm {i}^{\varSigma _0}_{\rho _0}\in I_{{\varGamma } \vdash {\theta }}^+$, there exists a deterministic $(|\nu (\mathrm {i})| ,m_j)$-automaton $\mathcal {A}_j$ of type $\theta$ with initial register assignment $\rho _0$ such that , where $P_{{\varGamma } \vdash {\theta }}^{ \mathrm {i}^{\varSigma _0}}$ is the set of plays over $\llbracket {\varGamma } \vdash {\theta } \rrbracket$ that start from $\mathrm {i}^{\varSigma _0}$.

### Reduction of inclusion into emptiness

The aim of this section is to establish the following result.

#### Lemma 30

Let ${\varGamma } \vdash {\mathbb {C}_1,\mathbb {C}_2:\theta }$ be -terms in canonical form. For each $j=\mathrm {i}^{\varSigma _0}_{\rho _0}\in I_{{\varGamma } \vdash {\theta }}^+$, there exists a deterministic $(|\nu (\mathrm {i})| ,n_j)$-automaton $\mathcal {B}_j$ with initial register assignment $\rho _0$ such that ${L}(\mathcal {B}_j)=\emptyset$ iff .

Suppose ${\varGamma } \vdash {\mathbb {C}_1,\mathbb {C}_2:\theta }$ be -terms in canonical form and let $\mathcal {A}_1$ and $\mathcal {A}_2$ be the automata representing their respective semantics for a given initial move $\mathrm {i}^{\varSigma _0}$. We construct an automaton $\mathcal {A}'$ such that:The idea behind the construction of $\mathcal {A}'$ is the following. We arrange so that the automaton behaves as a product automaton for $\mathcal {A}_1$ and $\mathcal {A}_2$, i.e. an automaton accepting their common strings (with possible extensions to stores on each side). The constructed automaton has the additional feature that, for each transition of $\mathcal {A}_1$, it checks whether the transition can be replicated by $\mathcal {A}_2$. If it can, then the automaton continues behaving as a product automaton. If the transition cannot be replicated then the automaton switches to *divergence mode* where it behaves as $\mathcal {A}_1$. The automaton accepts just if it reaches a final state in the divergence mode. The latter is justified by the fact that, in such a case, the automaton has detected a string in $\mathcal {A}_1$ which $\mathcal {A}_2$ cannot replicate and which leads to a complete play of $\llbracket \mathbb {C}_1 \rrbracket$.

Suppose now each $\mathcal {A}_i$ is an $(n_{\mathsf {r}},n_i)$-automaton given by $\mathcal {A}_i = \langle Q_i,q_{0i},\rho _{0i},\delta _i,F_i\rangle$ and assume that $\rho _{01}=\rho _{02}$. We first construct a generalised $(n_{\mathsf {r}},n_1,n_2)$-automaton $\mathcal {A}=\langle Q,q_0,\rho _0,\delta ,F\rangle$ that operates equivalently to our target automaton $\mathcal {A}'$ described above. In particular, we set:

$$\begin{aligned} Q=Q_1\cup (Q_{1O}\times Q_{2O})\cup (Q_{1P}\times Q_{2P}),\;\; q_0=(q_{01},q_{02}),\;\; \rho _0=\langle \rho _{01},\rho _{02}\rangle ,\;\; F=F_1, \end{aligned}$$

with $Q_N=(Q_{1O}\times Q_{2O})\cup (Q_{1P}\times Q_{2P})$, $Q_D=Q_1$ and $\mathsf {div}$ the first projection. For each $(q_1,q_2)\in Q$ we include in $\delta$ precisely the following transitions.

- If $q_i\xrightarrow {\pi _i}q_i'$, for $i=1,2$, then $(q_1,q_2)\xrightarrow {\pi _1,\pi _2}(q_1',q_2')$. Otherwise, if $q_1\xrightarrow {\pi }q_1'$ then $(q_1,q_2)\xrightarrow {\pi ,\checkmark }(q_1',q_2)$, and if $q_2\xrightarrow {\pi }q_2'$ then $(q_1,q_2)\xrightarrow {\checkmark ,\pi }(q_1,q_2')$.
- If $$q_i\xrightarrow {\nu X_i.(\ell _i,t_i,\phi _i)^{S_i}}q_i'$$, $$i=1,2$$, then $$(q_1,q_2)\xrightarrow {\nu X_1.(\ell _1,t_1,\phi _1)^{S_1},\nu X_2.(\ell _2,t_2,\phi _2)^{S_2}}(q_1',q_2')$$.
- If $$q_1\xrightarrow {\nu X_1.(\ell _1,t_1,\phi _1)^{S_1}}q_1'$$ then $$q_1\xrightarrow {\nu X_1.(\ell _1,t_1,\phi _1)^{S_1},\checkmark }q_1'$$.
- If $$q_1\xrightarrow {\pi _1}q_1'$$ then $$q_1\xrightarrow {\pi _1,\emptyset }q_1'$$.

#### Lemma 31

For $\mathbb {C}_1,\mathbb {C}_2,\mathcal {A}_1,\mathcal {A}_2$ and $\mathcal {A}$ as above:


#### Proof

Suppose $w_{\mathcal {A}}\in L(\mathcal {A})$. By construction of $\mathcal {A}$, the accepting run for $w_{\mathcal {A}}$ yields an accepting run for $w_1$ in $\mathcal {A}_1$ via first projection. By Lemma 29, we have . Consider $\mathrm {i}^{\varSigma _0} w \in P_{{\varGamma } \vdash {\theta }}^{ \mathrm {i}^{\varSigma _0}}$ that extends $\mathrm {i}^{\varSigma _0} w_{\mathcal {A}}$. We have $\mathrm {i}^{\varSigma _0} w \in \mathsf {ext}(\mathrm {i}^{\varSigma _0}w_1)$ and thus . On the other hand, via the second projection of the accepting run for $w_{\mathcal {A}}$ up to the point of switching to divergence mode, we obtain a run of $\mathcal {A}_2$ which, however, may only be resumed in $\mathcal {A}_2$ by a different (up to extension) P-move than that required by $w_{\mathcal {A}}$. Hence, $\mathrm {i}^{\varSigma _0} w\notin \llbracket {\varGamma } \vdash {\mathbb {C}_2:\theta } \rrbracket$. Conversely, let  and let $\mathrm {i}^{\varSigma _0}w'xy$ be its least prefix that appears in $\llbracket {\varGamma } \vdash {\mathbb {C}_1:\theta } \rrbracket {\setminus }\llbracket {\varGamma } \vdash {\mathbb {C}_2:\theta } \rrbracket$. By construction, our automata are closed under legal O- to P-transitions, so $\mathcal {A}_2$ must accept (a representation of) $\mathrm {i}^{\varSigma _0}w'x$ and fail to process *y*. On the other hand, $\mathcal {A}_1$ will be able to process a whole representation of $\mathrm {i}^{\varSigma _0}w$. Thus, $\mathcal {A}$ will operate as product automaton until $\mathrm {i}^{\varSigma _0}w'x$ and then enter divergence mode and continue as $\mathcal {A}_1$. Consequently, $\mathcal {A}$ will accept some $\mathrm {i}^{\varSigma _0}\tilde{w}$ such that $\mathrm {i}^{\varSigma _0} w\in \mathsf {ext}(\mathrm {i}^{\varSigma _0}\tilde{w})$, i.e. $L(\mathcal {A})\ne \emptyset$. $\square$

Determinacy extends to generalised automata in the obvious way: an automaton is deterministic if its configuration graph is. The notion of strong determinacy extends the following manner. By construction, the automaton $\mathcal {A}$ above is strongly deterministic.

#### Definition 32

Let $\mathcal {A}=\langle Q,q_0,\rho _0,\delta ,F\rangle$ be a generalised $(n_{\mathsf {r}},n_1,n_2)$-automaton. We say that $\mathcal {A}$ is *strongly deterministic* if:

- for each $q\in Q_{P}$ there is at most one transition out of *q* (i.e. $|\delta \upharpoonright \{q\}|\le 1$), and if $(q,z_1,z_2,q')\in \delta$ with either of $z_1,z_2$ of the form $\nu X.(\ell ,t,\phi )^S$ then $|\delta \upharpoonright \{q'\}|\le 1$ and in particular $q'$ may only have an outgoing transition $(q',z_1',z_2',q'')$ such that $z_i'\in \mathsf {Mix}$ iff $z_i\not =\checkmark$ and, for all $(q'',z_1'',z_2'',q''')\in \delta$, $z_1'',z_2''\notin \mathsf {Mix}$;
- for each $q\in Q_{O}$ and $(q,z_{i1},z_{i2},q_i)\in \delta$ with $z_{i1},z_{i2}\not \in \mathsf {Mix}$, $i=1,2$,
  - if $z_{11}\sim _\alpha z_{21}$ and $z_{12}\sim _\alpha z_{22}$ then $z_{11}=z_{21}$, $z_{12}=z_{22}$ and $q_1=q_2$;^8^
  - if $$z_{11},z_{12}\not =\checkmark $$ then $$z_{21},z_{22}\not =\checkmark $$;
  - of $z_{11}=\checkmark \not =z_{12}$ and $z_{21}\not =\checkmark =z_{22}$ then $z_{12}$ and $z_{21}$ contain different tags;
- for each $(q,z_1,z_2,q')\in \delta$, if either of $z_1,z_2$ is of the form $\nu X.(\ell ,t,\phi )^S$ then *X* is contained in $\mathsf {clo}(S,X_{\mathsf {Av}})$ where $X_{\mathsf {Av}}=(\mathsf {dom}(S){\setminus } X)\cup \{j\ |\ \ell ={\texttt {R}}_{j}\}$.

#### Lemma 33

If $\mathcal {A}$ is strongly deterministic then it is deterministic.

#### Proof

Again, cases with P- to O-transitions and transitions with rearrangements are taken care of by the first condition above. The case of O- to P-transition in one component follows from the second condition as in Lemma 25, using the first and last subcases of the second condition above. Finally, if such transitions happen in both components and induce, say, $\hat{q}\xrightarrow {(l,t)^{\varSigma }}\hat{q}_i$, $i=1,2$, where $\hat{q}$ has state *q*, let these be combination of transitions with labels $(l,t)^{\varSigma _{11}}$ and $(l,t)^{\varSigma _{12}}$, and of $(l,t)^{\varSigma _{21}}$ and $(l,t)^{\varSigma _{22}}$ respectively (here the second index specifies the component). We have $\varSigma =\varSigma _{11}\cup \varSigma _{12}=\varSigma _{21}\cup \varSigma _{22}$. Consider the associated $\nu X_{ij}.(\ell ,t,\phi _{ij})^{S_{ij}}$, for $i,j=1,2$, and in particular the ordinary transitions $(q,\nu X_{i1}.(\ell ,t,\phi _{i1})^{S_{i1}},q_1)$. Since they are both accepting from $\hat{q}$, it must be that $\phi _{11}=\phi _{21}$. Moreover, as $\varSigma _{11}$ and $\varSigma _{21}$ agree on their common names, $S_{11}$ and $S_{21}$ may only disagree on $X_{11},X_{21}$. But, by frugality, the latter are reachable from the indices of available registers and therefore $S_{11}=S_{21}$, modulo permutation of fresh indices. Thus, $\nu X_{11}.(\ell ,t,\phi _{11})^{S_{11}}\sim _\alpha \nu X_{21}.(\ell ,t,\phi _{21})^{S_{21}}$ and, similarly, $\nu X_{12}.(\ell ,t,\phi _{12})^{S_{12}}\sim _\alpha \nu X_{22}.(\ell ,t,\phi _{22})^{S_{22}}$. By strong determinacy we get $q_1=q_2$ and thus $\hat{q}_1=\hat{q}_2$. $\square$

The operation of a generalised automaton can be faithfully simulated by a corresponding ordinary automaton. More precisely, we can show that from a given generalised automaton $\mathcal {A}$ we can effectively construct a *bisimilar* ordinary one.

Let $G_1,G_2$ be labelled directed graphs with nodes selected from sets of *configurations* $\hat{Q}_1,\hat{Q}_2$ respectively, and labels selected from the set $\{\epsilon \}\cup (\mathcal {L}\times \mathbb {T}\times \mathsf {Sto})$. Moreover, let each $G_i$ have initial configuration $\hat{q}_{0i}$ and *final* configurations $\hat{Q}_{iF}$. We say that a relation $\mathcal {R}\subseteq \hat{Q}_1\times \hat{Q}_2$ is a ***simulation*** if, for all $\hat{q}_1\mathcal {R}\hat{q}_2$:

- if $$\hat{q}_1\in \hat{Q}_{1F}$$ then $$\hat{q}_2\in \hat{Q}_{2F}$$,
- if $\hat{q}_1\xrightarrow {l}_{G_1}\hat{q}_1'$, some $l\in \{\epsilon \}\cup (\mathcal {L}\times \mathbb {T}\times \mathsf {Sto})$, then $\hat{q}_2\xrightarrow {l}_{G_2}\hat{q}_2'$ for some $\hat{q}_1'\mathcal {R}\hat{q}_2'$.

We say that $\mathcal {R}$ is a ***bisimulation*** if both $\mathcal {R}$ and $\mathcal {R}^{-1}$ are simulations. Moreover, $G_1$ and $G_2$ are ***bisimilar***, written $G_1\sim G_2$, if there is a bisimulation $\mathcal {R}$ such that $\hat{q}_{01}\mathcal {R}\hat{q}_{02}$.

In particular, we say that $\mathcal {A}$ and $\mathcal {A}'$ are bisimilar, written $\mathcal {A}\sim \mathcal {A}'$, if their configuration graphs are bisimilar.

#### Lemma 34

Let $\mathcal {A}$ be a $(n_{\mathsf {r}},n_1,n_2)$-automaton and set $n'=n_1+n_2-n_{\mathsf {r}}$. We can effectively construct a $(n_{\mathsf {r}},n')$-automaton $\mathcal {A}'$ such that $\mathcal {A}\sim \mathcal {A}'$. Moreover, if $\mathcal {A}$ is strongly deterministic then so is $\mathcal {A}'$.

The construction of $\mathcal {A}'$ is presented in Appendix B. Combining Lemmata 31 and 34, and using the fact that bisimilarity implies language equivalence, we obtain the following.

#### Lemma 35

Let ${\varGamma } \vdash {\mathbb {C}_1,\mathbb {C}_2:\theta }$ be -terms in canonical form. For each $j=\mathrm {i}^{\varSigma _0}_{\rho _0}\in I_{{\varGamma } \vdash {\theta }}^+$, there exists a deterministic $(|\nu (\mathrm {i})| ,n_j)$-automaton $\mathcal {A}_j'$ with initial register assignment $\rho _0$ such that ${L}(\mathcal {A}_j')=\emptyset$ if and only if .

Lemma 30 then follows as a corollary.

### Emptiness for fresh pushdown register automata

Returning to Lemma 30, note that, although $I_{{\varGamma } \vdash {\theta }}^+$ is an infinite set, there exists a finite subset $J\subseteq I_{{\varGamma } \vdash {\theta }}^+$ such that $\{\mathcal {A}_j\}_{j\in J}$ already captures , because up to name-permutation there are only finitely many initial moves. Consequently, we only need finitely many of them to check whether . By Lemma 30, to achieve this we need to be able to decide the emptiness problem for $(n_{\mathsf {r}},n)$-automata.

To show decidability, we translate $(n_{\mathsf {r}},n)$-automata into an extended variant of pushdown register automata [7] (PDRA) over infinite alphabets. They are similar to $(n_{\mathsf {r}},n)$-automata in that they are equipped with registers and a stack. However, there are a few differences.

- PDRA can only process one name in a computational step, while $(n_{\mathsf {r}},n)$-automata read a label, a tag and a store in a single step. This can easily be overcome by decomposing transitions of our automata into a bounded number of steps (the existence of the bound follows from the fact that symbolic stores in our transition function are bounded).
- All registers in PDRA must be full, while $(n_{\mathsf {r}},n)$-automata admit empty registers. This difference can be compensated by populating registers with dummy names, while storing information about which register is deemed to be empty in the finite state.
- $(n_{\mathsf {r}},n)$ allow for rearrangements of registers in a single $\epsilon$-step. Again this can be decomposed into a sequence of $\epsilon$-transitions of an PDRA by using the stack.
- $(n_{\mathsf {r}},n)$ have the ability to create *globally* fresh names (guaranteed not to have been encountered in the whole computational run), while PDRA can only create *locally* fresh names (through the so-called reassignment), which are guaranteed not to occur in the present register assignment. This discrepancy cannot be dealt with easily and we provide a separate argument why the emptiness problems for the extension of PDRA with (globally) fresh-name generation remains decidable.

We start off with a generalisation of pushdown register automata [7] to data words. That is to say, in a non-epsilon step, the automaton reads a pair consisting of a tag (taken from a finite set of tags) and a value, which comes from the infinite alphabet. The latter will often be referred to as a *name*. Such pairs are also pushed on the stack. Decidability of emptiness in absence of fresh-name generation was already shown in [7] in the tag-free case, but the generalisation to tags is rather cosmetic, since they can be emulated with fixed names. Let $\varSigma$ be an infinite alphabet and $\mathcal {T}$ a finite set of tags with a distinguished bottom-of-stack tag $\bot$.

#### Definition 36

An *r*-register pushdown automaton (PDRA) $\mathcal {A}$ over $(\varSigma ,\mathcal {T})$ is a tuple $\langle Q,q^{ in },u,\rho ,\mu ,F \rangle$, where

- *Q* is a finite set of *states*;
- $q^{ in }\in Q$ is the *initial state*;
- $u:\{1,\cdots ,r\}\rightarrow \varSigma$ is an injection, called the *initial assignment*;
- $\rho : Q\rightarrow \{1,\cdots , r\}$ is a partial function called the *reassignment*;
- $\mu$ is the *transition relation*, which is a mapping from
  $$\begin{aligned} Q\times ((\mathcal {T}\times \{1,\cdots ,r\}) \cup \{\epsilon \})\times (\mathcal {T}\times \{1,\cdots ,r\}) \end{aligned}$$
  to finite subsets of $Q\times (\mathcal {T}\times \{1,\cdots ,r\})^*$;
- $F\subseteq Q$ is the set of *final states*.

A *configuration* is a triple (*q*, *R*, *S*) such that $q\in Q$, $R:\{1,\cdots ,r\}\rightarrow \varSigma$ is injective and $S \in (\mathcal {T}\times \varSigma )^*$. The last component represents the stack content (the leftmost symbol stands for the top of the stack). A configuration is *initial* if $q=q^{ in }$, $R=u$ and $S=[(\bot ,u(r))]$. A configuration is *final* if $q\in F$. A *generalised run* of $\mathcal {A}$ is a sequence $C_0\mathop {\rightarrow }\limits ^{x_1} \ldots \mathop {\rightarrow }\limits ^{x_k} C_{k}$ such that each $C_j =(q_i,R_i,S_i)$ ($0\le j\le k$) is a configuration, $q_0=q^{ in }$, $S_0=[(\bot ,R_0(r))]$, each $x_j \in \{\epsilon \}\cup (\mathcal {T}\times \varSigma )$ ($1\le j\le k$), and each $C_{j}$ ($0<j\le k$) is obtained from $C_{j-1}$ and $x_{j}$ according to $\mu$. Note that in generalised runs $R_0$ is left unspecified. This will make working with them slightly simpler, because they will be closed under bijective renamings. A *run* is simply a generalised run such that $C_0$ is initial. In an *accepting run* we also have $q_k\in F$.

We shall say that two generalised runs are *related* if they are of the same length and the corresponding steps rely on the same elements of $\mu$. Consequently, related generalised runs differ only in the names they involve (respective states and tags must be the same). We will be interested in characterizing generalised runs that are related to each other. Below we introduce several definitions that will make this possible.

Suppose $\mathcal {R}$ is a generalised run of length *k*. Let us assign *timestamps* from the set $\{1,\cdots , r+k\}$ to all occurrences of names in $\mathcal {R}$. They will indicate at which step the names were introduced into the run. Note that this can happen only in two ways: via the initial assignment in $C_0$ or reassignment through $\rho$. In other cases timestamps will be inherited from previous steps. Thus we can associate timestamps with occurrences of names in generalised runs as follows:

- the occurrence of $R_0(i)$ in $R_0$ is timestamped with *i* for each $1\le i\le r$, the occurrence of $R_0(r)$ in $S_0$ is timestamped with *r*;
- if a name is generated in step $1\le j\le k$ through reassignment, its occurrence in $R_j$ gets timestamp $r+j$, otherwise occurrences of names in registers inherit timestamps from preceding configurations;
- a name that was just pushed on the stack inherits the timestamp it had in the register before being pushed, the timestamps of other names on the stack are inherited from preceding configurations.

In what follows we shall refer to timestamps using notation such as $t_{R_j(i)}$ or $t_{ top (S_j)}$.

Let us write $\mathbb {T}\subseteq \{1,\cdots , r+k\}$ for the set of all timestamps used to mark occurrences of names of $\mathcal {R}$.

#### Remark 37

Note that, due to the stack discipline, whenever the same names are present on the stack in a configuration of a run, the associated sequence of timestaps, from top to bottom, must be non-increasing. Additionally, if the same name occurs in a register, its timestamp will not be smaller than the timestamps of occurrences of the same name on the stack.

We can replace all occurrences of names in $\mathcal {R}$ with their timestamps to obtain what we shall call a *symbolic run*: $C_0^ sym \mathop {\rightarrow }\limits ^{x_1^{ sym }}\cdots \mathop {\rightarrow }\limits ^{x_k^ sym } C_k^{ sym }$. Recall that states and tags will remain the same as in $\mathcal {R}$. Observe the following fact.

#### Lemma 38

Two generalised runs are related if and only if the corresponding symbolic runs are the same.

Next we characterize assignments $\alpha :\mathbb {T}\rightarrow \varSigma$ (of names to timestamps), which can be used to convert a symbolic run generated from $\mathcal {R}$ into a generalised run related to $\mathcal {R}$.

- First, the initial register assignment must be injective: $\alpha (i)\ne \alpha (j)$ for $1\le i<j\le r$.
- The second set of constraints comes from reassignment steps, where it must be ensured that the reassigned name is different from those currently present in registers: if $\rho (q_i)$ ($0\le i < k$) is defined then we require that $\alpha (t_{R_{i+1}(\rho (q_i))})\ne \alpha (t_{R_i (j)})$ for any $1\le j\le r$.
- The third set of constraints is induced by popping during transitions: the name on top of the stack must match the content of a suitable register, as specified by $\mu$: if the passage from $q_i$ to $q_{i+1}$ ($0\le i <k$) relies on an element of $\mu (q_i, (t_1,i_1), (t_2,i_2))$ or $\mu (q_i, \epsilon , (t_2,i_2))$ then $\alpha (t_{ top (S_i)}) = \alpha (t_{R_{i+1}(i_2)})$.

Altogether, above we have extracted from $\mathcal {R}$ a set of conditions characterizing runs related to $\mathcal {R}$, in terms of which names introduced into a run must be equal or unequal.

#### Lemma 39

Generalised runs related to $\mathcal {R}$ are in 1–1 correspondence with $\alpha {:}\mathbb {T}\rightarrow \varSigma$ satisfying the above constraints.

Below we single out a special family of such maps. Intuitively, we shall focus on generalised runs in which as many names as possible are used.

Let us define $=_\mathcal {R}$ to be the smallest equivalence relation such that if ‘$\alpha (i)=\alpha (j)$’ belongs to the third set of constraints we have $i=_\mathcal {R}j$. $\alpha ': \mathbb {T}\rightarrow \varSigma$ will be called *distinctive* if, for all $i,j\in \mathbb {T}$, we have $\alpha '(i)=\alpha '(j)$ if and only if $i=_\mathcal {R}j$.

#### Remark 40

Note that every distinctive $\alpha '$ satisfies the first two kinds of constraints and, hence, gives rise to a generalised run. By definition distinctive maps $\alpha '$ exist and are determined uniquely up to name-permutation. In particular, there exists a distinctive $\alpha '$ that is compatible with the initial register assignment in $\mathcal {R}$.

The following property of distinctive maps will play a role in a future argument.

#### Lemma 41

Let $\alpha '$ be distinctive, $i\in \mathbb {T}$ and $k_i=\min \,\{j\,|\, i=_\mathcal {R}j\}$. Then for all $j < k_i$ we have $\alpha '(j)\ne \alpha '(k_i)=\alpha '(i)$. In other words, $\alpha '(k_i)$ is fresh.

#### Proof

Take $j<k_i$. By definition of $k_i$, it is not the case that $i =_\mathcal {R}j$. Because $\alpha '$ is distinctive, we must have $\alpha '(j)\ne \alpha '(k_i)$. On the other hand, $k_i=_\mathcal {R}i$, so by distinctiveness $\alpha '(k_i)=\alpha '(i)$. $\square$

#### Definition 42

A fresh PDRA (FPDRA) $\mathcal {A}$ over $(\varSigma ,\mathcal {T})$ is defined in the same way as a PDRA except that the (partial) reassignment function has the form $\rho :Q\rightarrow \{1,\cdots ,r\}\times \{L,G\}$. Whenever $\pi _2(\rho (q))= L$ (locally fresh), $\mathcal {A}$ must generate a name that is currently not present in registers. If $\pi _2(\rho (q))=G$ (globally fresh) then the name must in addition not have occurred before in the present run (in particular, it will not occur on the stack).

We shall show that the emptiness problem for FPDRA is decidable by referring to the analogous result for PDRA [7]. Let $\mathcal {A}=\langle \, Q,q^ in ,u,\rho ,\mu ,F \,\rangle$ be a FPDRA over $(\varSigma ,\mathcal {T})$. Next we are going to define an PDRA $\mathcal {A}'=\langle \, Q',q^{ in '},u', \rho ',\mu ', F' \,\rangle$ such that there exists an accepting run of $\mathcal {A}$ iff there exists one for $\mathcal {A}'$.

$\mathcal {A}'$ will mimic steps made by $\mathcal {A}$ except that it will not be able to generate globally fresh names, so it will use locally fresh ones instead. In addition, $\mathcal {A}'$ will maintain information about such names by flagging registers and stack elements.

- A flagged register signifies the fact that in $\mathcal {A}$ the generated name would be different from any name currently occurring on the stack.
- A flagged name on the stack means that in $\mathcal {A}$ all names underneath it would be different.

Register flags will be stored inside the extended state, flags for names on the stack will be assigned by appending *F* to the associated tag. The way the flags are managed is described below.

- Whenever a locally fresh name is generated instead of a globally fresh name, the corresponding register will remain flagged as long as its content is not pushed on the stack.
- When a sequence of names is pushed on the stack, for each name that comes from a flagged register, we flag the rightmost (deepest) of its occurrences in the sequence. The registers in question become untagged.
- A name can be popped only if it occurs in an unflagged register. If the name is flagged on the stack, we then add a flag to the register. Note that we do not allow $\mathcal {A}'$ to follow $\mathcal {A}$, if this would entail popping a symbol that occurs in a flagged register.

Next we present the construction formally. $\mathcal {A}'$ will be an PDRA over $(\varSigma , \mathcal {T}')$, where:To define $\mu '$, we use $t_1, t_2$ to range over $\mathcal {T}$ and $t_2^f, c_1^f, \cdots , c_n^f$ for elements of $\mathcal {T}'$. Whenever we use $t^f$ to refer to elements of $\mathcal {T}'$, by dropping the superscript and writing *t* we mean to refer to the underlying tag from $\mathcal {T}$.

$$\begin{aligned} ((p,Y), (c_1^f,j_1)\cdots (c_n^f,j_n))\in \mu '((q,X),(t_1^f, i_1), (t_2^f, i_2)) \end{aligned}$$

if $(p,(c_1,j_1)\cdots (c_n,j_n))\in {\mu }(q,(t_1, i_1), (t_2, i_2))$ and $i_2\not \in X$, $Y=X'{\setminus } \{j_1,\cdots ,j_n\}$ where

$$\begin{aligned} X'= X\cup \{\pi _1 (\rho (q))\,\,|\,\, \pi _2(\rho (q))=G\} \cup \{i_2 \,\,|\,\, t_2^f\in \mathcal {T}\times \{F\}\}, \end{aligned}$$

and, for all $i=1,\cdots , n$,

$$\begin{aligned} c_i^f=\left\{ \begin{array}{ccl} (c_i,F) &{}\quad &{} j_i\in X', \,\, \,\forall _{i<l\le n}\,\, j_l\ne j_i\\ c_i &{}&{} \text {otherwise}. \end{array}\right. \end{aligned}$$

#### Lemma 43

$\mathcal {A}$ has an accepting run if and only if $\mathcal {A}'$ has one.

#### Proof

Suppose there exists an accepting run of $\mathcal {A}$. The same run is then accepting for $\mathcal {A}'$. This is because whenever a register is flagged in $\mathcal {A}'$, it will indeed contain a name not present on the stack, and if a name is flagged on the stack the name will not occur below. Consequently, whenever a step of $\mathcal {A}$ depends on matching a name on top of the stack with a register, the corresponding register in $\mathcal {A}'$ will not be flagged. Thus, every step of $\mathcal {A}$ can be simulated by $\mathcal {A}'$.

Suppose we have an accepting run $\mathcal {R}$ of $\mathcal {A}'$. As $\mathcal {A}'$-transitions are transitions of $\mathcal {A}$ enriched with some information, it suffices to show that the locally fresh names generated instead of globally fresh names are globally fresh. This need not be the case for $\mathcal {R}$, but will show how to rename $\mathcal {R}$ so that another $\mathcal {A}'$-run emerges, which does enjoy the property.

Let us consider a step of $\mathcal {R}$ in which a locally fresh name is used as a substitute for a globally fresh one. Let *t* be the timestamp assigned to that name. We show that $t'=_\mathcal {R}t$ implies $t'\ge t$. For a start, we note that the ‘$\alpha (i)=\alpha (j)$’ constraints generated before time *t* concern *i*, *j* strictly smaller than *t*. We show that all the equational constraints that affect $[t]_{=_\mathcal {R}}$ later concern timestamps that are at least *t*. We analyze the history of the name timestamped *t* during the run.

- Unless the name is pushed immediately on the stack, it will stay flagged in registers for a number of steps. Thus no pops will rely on it. Hence no constraints using $\alpha (t)$ will be generated.
- After the name is pushed on the stack, its deepest occurrence in the push sequence will be flagged. The register will be unflagged but, as long as the flagged occurrence remains on the stack, all equational constraints that rely on the same name will involve timestamps greater than or equal to *t* (see Remark 37).
- When the flagged occurrence with timestap *t* is eventually popped (if at all), there must be a register containing the same name, but this occurrence will bear a timestamp $t'$ such that $t'\ge t$ and will become flagged. From then on the reasoning can be repeated for $t'$ and, since $t'\ge t$, we can conclude that future constraints affecting $[t']_{=_\mathcal {R}}=[t]_{=_\mathcal {R}}$ will not involve timestamps smaller than $t'$.

So, for each timestamp *t* corresponding to a globally fresh name in $\mathcal {A}$, we have $[t]_{=_\mathcal {R}} \subseteq [t,\infty )$. Consequently, by Remark 40, in every related distinctive run, names with that timestamp will indeed be different from all names used by the automaton earlier, i.e. they will be globally fresh. Moreover, there exists a distinctive run whose initial assignment is *u*. That run is thus also a run of $\mathcal {A}$ (after erasing the flag information). $\square$

The above result reduces the emptiness problem for FPDRA to the analogous problem for PDRA. As the latter is decidable [7], we obtain the following.

#### Lemma 44

The emptiness problem for FPDRA is decidable.

Finally, summing up, we obtain the desired decidability result.

#### Theorem 45

Program approximation (and thus program equivalence) is decidable for -terms.

#### Proof

Let ${\varGamma } \vdash {M_1,M_2:\theta }$ be -terms. By Lemma 2, they can be converted into canonical forms ${\varGamma } \vdash {\mathbb {C}_{M_1}, \mathbb {C}_{M_2}:\theta }$ such that  if and only if . By Lemma 30 and our observations at the beginning of Sect. 5.4, the problem of determining whether  holds can be reduced to the emptiness problem for a finite number of FPDRA, all effectively constructible from $\mathbb {C}_{M_1}, \mathbb {C}_{M_2}$. Because FPDRA emptiness is decidable by Lemma 44, program approximation is decidable for . $\square$

## Related and further work

In this paper we achieved a full characterisation of decidability of program equivalence in $\mathsf {GRef}$, a higher-order language with full ground storage. Moreover, for the decidable fragment that we identified, we devised a decidability procedure which builds on automata-based representations of terms.

The investigations into models and reasoning principles for storage have a long history. In this quest, storage of names was regarded by researchers as an indispensable intermediate step towards capturing realistic languages with dynamic-allocated storage, such as ML or Java. Relational methods and environmental bisimulations for reasoning about program equivalence in settings similar to ours were studied in [2, 5, 9, 16, 30, 31], albeit without decidability results. More foundational work included labelled transition system semantics [15] and game semantics [19, 24]. In both cases, it turned out that the addition of name storage simplified reasoning, be it bisimulation-based or game-semantic. In the former case, bisimulation was even unsound without full ground storage. In the latter case, the game model of integer storage [22] turned out more intricate (complicated store abstractions) than that for full ground or general storage [19, 24].

As for decidability results, we studied finitary Reduced ML (integer storage only) in [23], yet only judgements of the form ${\cdots , \beta \rightarrow \beta ,\cdots } \vdash {\beta }$ were tackled due to intricacies related to store abstractions (in absence of full ground storage, names cannot be remembered by programs). A closely related language, called RML [1] (integer storage but with bad references) was studied in [8, 14, 27], but no full classification has emerged yet. Interestingly, closed terms of first-order type become decidable in this setting [8], in contrast to $\mathsf {unit}\rightarrow \mathsf {unit}\rightarrow \mathsf {unit}$ for $\mathsf {GRef}$. Finally, the approach presented herein was pursued for Interface Middleweight Java [21] and implemented in the equivalence checker Conneqct [20]. We note that, in presence of higher-order references and boolean storage, even termination is undecidable [26].

Apart from the semantics and programming languages community, program equivalence has been extensively examined in the context of regression verification [4, 6, 10, 12, 18]. There, the focus is put on sound methods for proving that newer versions of the same code fragment do not introduce new behaviours, outside the code specification. In terminology used in this paper, the property that is being verified is program approximation. Regression verification is based on strong abstraction techniques which are sound for approximation, and is typically applied to ground languages and code (i.e. no higher-order functions). In the future, we would like to expand the use of game models for checking equivalence to larger language fragments, where equivalence in undecidable. In doing so, we would abandon decidability and restrict ourselves to sound verification routines, using similar abstraction techniques.

## A Canonical forms for $\mathsf {GRef}$

In this section we prove Lemma 2:
*Let* ${\varGamma } \vdash {M:\theta }$ *be an* $\mathsf {GRef}$-*term. There exists a* $\mathsf {GRef}$-*term* ${\varGamma } \vdash {\mathbb {C}_M:\theta }$ *in canonical form, effectively constructible from* *M*, *such that* ${\varGamma } \vdash {M\cong \mathbb {C}_M}$. We prove two auxiliary results first, both of which are special cases of Lemma 2.

### Lemma 46

Any identifier $x^\theta$ satisfies Lemma 2. Moreover, when $\theta \equiv \theta _1\rightarrow \theta _2$, the canonical form is of the form $\lambda y^{\theta _1}.\mathbb {C}$.

### Proof

Induction with respect to type structure. If $\theta$ is a base type, we can take $\mathbb {C}_{x^\mathsf {unit}}$ to be () and $\mathbb {C}_{x^\mathsf {int}}$ to be $\mathsf {case}(x^\mathsf {int})[0,\cdots , max ]$. $x^{\mathsf {ref}\, \gamma }$ is already in canonical form. For $\theta \equiv \theta _1\rightarrow \theta _2$ we use the $\eta$-expansion rule

$$\begin{aligned} x^{\theta _1\rightarrow \theta _2} \cong \lambda z^{\theta _1}. \mathsf {let}\,y^{\theta _2}=x z^{\theta _1}\,\mathsf {in}\,y \end{aligned}$$

and appeal to the inductive hypothesis for $z^{\theta _1}$ and $y^{\theta _2}$. This suffices for $\theta _1\equiv \mathsf {unit},\mathsf {ref}\, , {\theta _1'\rightarrow \theta _2'}$. For $\theta _1\equiv \mathsf {int}$ we additionally observe that

$$\begin{aligned} \begin{array}{c} \mathsf {let}\,y=x (\mathsf {case}(z)[0,\cdots , max ])\,\mathsf {in}\,y\\ \cong \\ \mathsf {case}(z)[\mathsf {let}\,y=x\,0\,\mathsf {in}\,y,\cdots ,\mathsf {let}\,y=x\, max \,\mathsf {in}\,y]. \end{array} \end{aligned}$$

$$\square $$

### Lemma 47

Suppose $\mathbb {C}_1, \mathbb {C}_2$ are canonical forms. Then $\mathsf {let}\,y^\theta =\mathbb {C}_1\,\mathsf {in}\,\mathbb {C}_2$, if typable, satisfies Lemma 2.

### Proof

Induction with respect to the structure of $\theta$.

- $\theta \equiv \mathsf {unit}$. Using the equivalences listed below, we reason by induction on the structure of $\mathbb {C}_1$, which can take one of the following shapes: (), $\mathsf {case}(x^\mathsf {int})[C_0,\cdots ,C_ max ]$ or $\mathsf {let}\,\cdots \,\mathsf {in}\,C$ 9. Note that free identifiers of type $\mathsf {unit}$ never occur in canonical forms, hence the first equivalence.
  $$\begin{aligned}\begin{array}{rcl} \mathsf {let}\,y=()\,\mathsf {in}\,\mathbb {C}_2 &{}\cong &{}\mathbb {C}_2\\ \mathsf {let}\,y=\mathsf {case}(x^\mathsf {int})[C_0,\cdots ,C_ max ]\,\mathsf {in}\,\mathbb {C}_2 &{}\cong &{} \mathsf {case}(x)[\mathsf {let}\,y=C_0\,\mathsf {in}\,\mathbb {C}_2,\cdots ,\mathsf {let}\,y=C_ max \,\mathsf {in}\,\mathbb {C}_2]\\ \mathsf {let}\,y=(\mathsf {let}\,\cdots \,\mathsf {in}\,C)\,\mathsf {in}\,\mathbb {C}_2&{}\cong &{} \mathsf {let}\,\cdots \,\mathsf {in}\,(\mathsf {let}\,y=C\,\mathsf {in}\,\mathbb {C}_2) \end{array}\end{aligned}$$
- $\theta \equiv \mathsf {int}$. We reason by induction on the structure of $\mathbb {C}_1$, which can take one of the following shapes: *i*, $\mathsf {case}(x^\mathsf {int})[C_0,\cdots ,C_ max ]$ or $\mathsf {let}\,\cdots \,\mathsf {in}\,C$. The last two cases are dealt with as before. For $\mathsf {let}\,y=i\,\mathsf {in}\,\mathbb {C}_2$, note that, whenever identifiers $y^\mathsf {int}$ occur freely in canonical forms, it is always as part of a subterm of the form $\mathsf {case}(y^\mathsf {int})[\mathbb {C}_0,\cdots ,\mathbb {C}_ max ]$. Given a canonical form $\mathbb {C}$ we write $\mathbb {C}^{y,i}$ for the canonical term obtained from $\mathbb {C}$ by replacing each of its subterms of the form $\mathsf {case}(y^\mathsf {int})[\mathbb {C}_0,\cdots ,\mathbb {C}_ max ]$ with $\mathbb {C}_i$ (i.e. by removing *y* and branches corresponding to $y\ne i$). Then
  $$\begin{aligned} \mathsf {let}\,y=i\,\mathsf {in}\,\mathbb {C}_2 \cong \mathbb {C}_2^{y,i}. \end{aligned}$$
- $\theta \equiv \mathsf {ref}\, \gamma$. We reason by induction on the structure of $\mathbb {C}_1$, which can take one of the following shapes: $x^{\mathsf {ref}\, \gamma }$, $\mathsf {case}(x)[C_0,\cdots ,C_ max ]$ or $\mathsf {let}\,\cdots \,\mathsf {in}\,C$. The last two inductive cases are dealt with as before. For $\mathsf {let}\,y=x^{\mathsf {ref}\, \gamma }\,\mathsf {in}\,\mathbb {C}_2$ we observe that $\mathbb {C}_2[x/y]$ is in canonical form and
  $$\begin{aligned} \mathsf {let}\,y=x^{\mathsf {ref}\, \gamma }\,\mathsf {in}\,\mathbb {C}_2\cong \mathbb {C}_2[x/y]. \end{aligned}$$
- $\theta \equiv \theta _1\rightarrow \theta _2$. As earlier, we reason by induction on the structure of $\mathbb {C}_1$, which can now take one of the following shapes: $\lambda x^\theta _1.C$, $\mathsf {case}(x^\mathsf {int})[C_0,\cdots ,C_ max ]$ or $\mathsf {let}\,\cdots \,\mathsf {in}\,C$. We can deal with the last two cases as before. The first one requires a separate argument, though.
  Suppose $\mathbb {C}_1\equiv \lambda x_1^{\theta _1}.C_1$. Let us substitute $\mathbb {C}_1$ for the rightmost occurrence of *y* in $\mathbb {C}_2$. This will create a non-canonical subterm in $\mathbb {C}_2$ of the form $\mathsf {let}\,x^{\theta _2}=(\lambda x_1^{\theta _1}.C_1) C\,\mathsf {in}\,C_2\equiv \mathsf {let}\,x^{\theta _2}=(\mathsf {let}\,x_1^{\theta _1}=C\,\mathsf {in}\,C_1)\,\mathsf {in}\,C_2$. By inductive hypothesis for $\theta _1$, $\mathsf {let}\,x_1^{\theta _1}=C\,\mathsf {in}\,C_1$ can be converted to canonical form, say, $C_3$. Consequently, the non-canonical subterm $\mathsf {let}\,x^{\theta _2}=(\lambda x_1^{\theta _1}.C_1) C\,\mathsf {in}\,C_2$ can be converted to $\mathsf {let}\,x^{\theta _2}=C_3\,\mathsf {in}\,C_2$, which — by inductive hypothesis for $\theta _2$ — can also be converted to canonical form. Thus, we have shown how to recover canonical forms after substitution for the rightmost occurrence of *y*. Because of the choice of the rightmost occurrence, the transformation does not involve terms containing other occurrences of *y*, so it will also decrease their overall number in $\mathbb {C}_2$ by one. Consequently, by repeated substitution for rightmost occurrences one can eventually arrive at a canonical form for $\mathsf {let}\,y^\theta =(\lambda x_1^{\theta _1}.C_1)\,\mathsf {in}\,\mathbb {C}_2$. $\square$

Now we are ready to prove Lemma 2.

### Proof

(Lemma 2) We proceed by induction on term structure.

- (), *i* are already in canonical form.
- For $\mathsf {case}(M)[N_0,\cdots ,N_ max ]$ we simply appeal to Lemma 47 for
  $$\begin{aligned} \mathsf {let}\,x^\mathsf {int}=\mathbb {C}_M\,\mathsf {in}\,\mathsf {case}(x)[\mathbb {C}_{N_0},\cdots ,\mathbb {C}_{N_ max }]. \end{aligned}$$
- For !*M* we apply Lemma 47 to
  $$\begin{aligned} \mathsf {let}\,x^{\mathsf {ref}\, \gamma }=\mathbb {C}_M\,\mathsf {in}\,(\mathsf {let}\,y^\gamma =!x\,\mathsf {in}\,\mathbb {C}_{y^\gamma }). \end{aligned}$$
- For $M:=N$, depending on whether *N*’s type is ${\mathsf {int}}$ or ${\mathsf {ref}\, \gamma }$, we appeal to Lemma 47 for
  $$\begin{aligned} \mathsf {let}\,y^\mathsf {int}=\mathbb {C}_N\,\mathsf {in}\,\mathsf {case}(y)[(x^{\mathsf {ref}\, \mathsf {int}}:=0);(),\cdots ,(x^{\mathsf {ref}\, \mathsf {int}}:= max );()] \end{aligned}$$
  or
  $$\begin{aligned} \mathsf {let}\,y^{\mathsf {ref}\, \gamma }=\mathbb {C}_N\,\mathsf {in}\,((x^{\mathsf {ref}\, \mathsf {ref}\, \gamma }:=y^{\mathsf {ref}\, \gamma }); ()) \end{aligned}$$
  to obtain $\mathbb {C}$ and then invoke the same lemma for $\mathsf {let}\,x=\mathbb {C}_M\,\mathsf {in}\,\mathbb {C}$.
- For $\mathsf {ref}(M)$, depending on whether *M*’s type is $\mathsf {int}$ or $\mathsf {ref}\, \gamma$, we invoke Lemma 47 for
  $$\begin{aligned} \mathsf {let}\,y^\mathsf {int}=\mathbb {C}_M\,\mathsf {in}\,\mathsf {let}\,x=\mathsf {ref}(0)\,\mathsf {in}\,\mathsf {case}(y)[(x:=0);x,\cdots ,(x:= max );x]. \end{aligned}$$
  or
  $$\begin{aligned} \mathsf {let}\,y^{\mathsf {ref}\, \gamma }=\mathbb {C}_M\,\mathsf {in}\,\mathsf {let}\,x^{\mathsf {ref}\, \mathsf {ref}\, \gamma }=\mathsf {ref}(y^{\mathsf {ref}\, \gamma })\,\mathsf {in}\,\mathbb {C}_x. \end{aligned}$$
- For $\mathsf {while}\,M\,\mathsf {do}\,N$, we note that $\mathsf {while}\,M\,\mathsf {do}\,N$ is equivalent to
  $$\begin{aligned} \mathsf {let}\,z=\mathsf {ref}(0)\,\mathsf {in}\,(z:=M; \mathsf {while}\,(!z)\,\mathsf {do}\,(N;z:=M)), \end{aligned}$$
  where *z* is fresh. We can find a canonical form for the latter term by appealing to the inductive hypothesis and previous lemmas. By the preceding case, we can obtain $\mathbb {C}_{z:=M}$. Using Lemma 47 we find $\mathbb {C}_{N;{z:=M}}$. Note that $\mathsf {while}\,(!z)\,\mathsf {do}\,\mathbb {C}_{N;z:=M}$ is then a canonical form. Next we can invoke Lemma 47 again for $\mathbb {C}_{z:=M}$ and $\mathsf {while}\,(!z)\,\mathsf {do}\,\mathbb {C}_{N;z:=M}$ to obtain $\mathbb {C}$. $\mathsf {let}\,z=\mathsf {ref}(0)\,\mathsf {in}\,\mathbb {C}$ is then a suitable canonical form for $\mathsf {while}\,M\,\mathsf {do}\,N$.
- For $\lambda x^\theta .M$ we can use $\lambda x^\theta .\mathbb {C}_M$.
- Finally, we handle application *MN*. By inductive hypothesis we obtain $\mathbb {C}_M$ which must take one of the following shapes: $\lambda x^\theta .C$, $\mathsf {case}(x)[C_0,\cdots ,C_ max ]$ or $\mathsf {let}\,\cdots \,\mathsf {in}\,C$. Using the equivalences below (and Lemma 47) we can then reason by induction on the possible structure of $\mathbb {C}_M$.
  $$\begin{aligned}\begin{array}{rcl} (\lambda x^\theta .C) N &{}\cong &{} \mathsf {let}\,x=\mathbb {C}_N\,\mathsf {in}\,C\\ (\mathsf {case}(x)[C_0,\cdots ,C_ max ])N &{}\cong &{} \mathsf {case}(x)[C_0 N,\cdots , C_ max N]\\ (\mathsf {let}\,\cdots \,\mathsf {in}\,C)N &{}\cong &{} \mathsf {let}\,\cdots \,\mathsf {in}\,CN \end{array} \end{aligned}$$
  $$\square $$

## B Reduction via spans

This section shows how to reduce generalised automata to ordinary ones. We shall achieve this via bisimulation equivalence, using a notion of correspondence between different name environments called *span*.

Let $\mathcal {A}=\langle Q,q_0,\rho _0,\delta ,F\rangle$ be a $(n_{\mathsf {r}},n_1,n_2)$-automaton and set $n'=n_1+n_2-n_{\mathsf {r}}$. We proceed to construct an $(n_{\mathsf {r}},n')$-automaton $\mathcal {A}'$ such that $\mathcal {A}\sim \mathcal {A}'$. The automaton will simulate $\mathcal {A}$ and in particular it will encode the pair of register assignments of the latter into a single one. For this, it will use an auxiliary environment component. Let $N_1=[n_{\mathsf {r}}+1,n_1]$, $N_2=[n_{\mathsf {r}}+1,n_2]$ and $N'=[n_{\mathsf {r}}+1,n']$. We call

$$\begin{aligned} (X_1,R,X_2)\in \mathcal {P}(N_1)\times \mathcal {P}(N_1\times N_2\times \{1,2\})\times \mathcal {P}(N_2) \end{aligned}$$

a *span* on $N_1,N_2$ if:

- $(i,j,z),(i',j',z')\in R$ implies that $i=i'\iff j=j'$, and $i=i'\implies z=z'$,
- $$\mathsf {dom}(R)=\{i\ |\ \exists j,z.\,(i,j,z)\in R\}\subseteq X_1$$ and $$\mathsf {cod}(R)=\{j\ |\ \exists i,z.\,(i,j,z)\in R\}\subseteq X_2$$.

We write $N_1\rightleftharpoons N_2$ for the set of spans on $N_1,N_2$. By abuse of notation, we write *R* for the whole of $(X_1,R,X_2)$, in which case we also use the notation $\mathsf {xdom}(R)=X_1$ and $\mathsf {xcod}(R)=X_2$. We also define a map $\overline{R}:[1,n']\rightarrow [1,n']$:

$$\begin{aligned} \overline{R} =&\ \{(i,i),(j^+,i)\ |\ (i,j,1)\in R\}\cup \{(i,i)\ |\ i\in (N_1{\setminus }\mathsf {dom}(R))\cup [1,n_{\mathsf {r}}]\}\\&\,\cup \{(i,j^+),(j^+,j^+)\ |\ (i,j,2)\in R\}\cup \{(j^+,j^+)\ |\ j\in N_2{\setminus }\mathsf {cod}(R)\} \end{aligned}$$

The notation extends to other domains containing indices, e.g. for labels: $\overline{R}({\texttt {R}}_{i})={\texttt {R}}_{\overline{R}(i)}$ and $\overline{R}(\ell )=\ell$ if $\ell$ is not a register.

The role of a span is to allow us to simulate a pair of register assignments in a single one. $(X_1,R,X_2)$ represents a pair of assignments where the first one has domain $X_1$ and the second one $X_2$. Moreover, *R* relates the common names: e.g. if $(i,j,z)\in R$ then register *i* in the first assignment contains the same name, say *a*, as register *j* in the second one. Finally, the index *z* specifies in which part of the simulating assignment does *a* really occur. For example, the pair of register assignments (suppose $n_{\mathsf {r}}=0$ for simplicity, and $n_1=n_2=3$),

$$\begin{aligned} \rho _1 = \{(2,b),(3,a)\},\,\,\,\, \rho _2 = \{(1,d),(2,a),(3,e)\} \end{aligned}$$

can be represented by the single assignment

$$\begin{aligned} \rho = \{(2,b),(3,a),(4,d),(6,e)\} \end{aligned}$$

with related span $R=(\{2,3\},\{(3,2,1)\},\{1,2,3\})$. This is because $\rho _1$ has domain $\{2,3\}$, $\rho _2$ has domain $\{1,2,3\}$, and the only common name is $a=\rho _1(3)=\rho _2(2)$, and is stored in the first half of $\rho$. The map $\overline{R}=\{(1,1),(2,2),(3,3),(4,4),(5,3),(6,6)\}$ then allows us to reconstruct $\rho _1,\rho _2$ by:

$$\begin{aligned} \langle \rho _1,\rho _2\rangle = \{(2,b),(3,a),(4,d),(5,a),(6,e)\} =\rho \circ \overline{R} \end{aligned}$$

In effect, $\overline{R}$ maps each index of the extended register $\langle \rho _1,\rho _2\rangle$ to its “real” position in $\rho$.

If $\rho _1,\rho _2$ are register assignments of size $n_1,n_2$ respectively and with common $[1,n_{\mathsf {r}}]$-part then we can obtain the span which accommodates all common names to the left by:

$$\begin{aligned} \rho _1\leftrightarrow \rho _2 = (\mathsf {dom}(\rho _1)\cap N_1,\{(i,j,1)\ |\ i\in N_1,j\in N_2,\rho _1(i)=\rho _2(j)\},\mathsf {dom}(\rho _2)\cap N_2) \end{aligned}$$

The single register assignment which simulates $\rho _1$ and $\rho _2$ via $\rho _1\leftrightarrow \rho _2$ is then given by $\rho =\langle \rho _1,\rho _2\rangle \circ \{(i,i)\ |\ \overline{(\rho _1\leftrightarrow \rho _2)}(i)=i\}$.

If $R:N_1\rightleftharpoons N_2$ and $\pi _1,\pi _2\in \mathsf {Mix}$ then we define the update of *R* with respect to the two (componentwise) rearrangements $\pi _1$ and $\pi _2$:

$$\begin{aligned} R[\pi _1,\pi _2]&= (\{i\ |\ \pi _1(i)\in \mathsf {xdom}(R)\},R',\{j\ |\ \pi _2(j)\in \mathsf {xcod}(R)\}) \\ R'&= \{(i,j,z)\ |\ (\pi _1(i),\pi _2(j),z)\in R\} \end{aligned}$$

Moreover, we extend the shift notation componentwise to other constructions, e.g. for symbolic stores: $S^+=\{(i^+,{\texttt {R}}_{j^+})\ |\ (i,{\texttt {R}}_{j})\in S\}\cup \{(i^+,j)\ |\ (i,j)\in S\}$. Finally, if $\pi ,\pi '\in \mathsf {Mix}$ then we let $\pi [\pi ']=\pi '\cup \{(i,\pi (i))\ |\ i\notin \mathsf {dom}(\pi ') \}$.

For $\mathcal {A}$ given as above, we define an $(n_{\mathsf {r}},n')$-automaton $\mathcal {A}'=\langle Q',q_0',\rho _0',\delta ',F'\rangle$ by:

$$\begin{aligned} Q'&= Q\times (N_1\rightleftharpoons N_2)\times \mathsf {SSto}\\ q_0'&= (q_{0},R_0,\emptyset )&R_0&= \pi _1(\rho _{0})\leftrightarrow \pi _2(\rho _{0})\\ \rho _0'&= \rho _0\circ \{(i,i)\ |\ R_0(i)=i \}&F'&= \{ (q,R,S)\in Q'\ |\ q\in F\} \end{aligned}$$

The crux of the construction is the definition of $\delta '$. The automaton will simulate the generalised automaton using one copy of each common name. *R* will be used as a record of common/private names between the two register components of $\mathcal {A}$, and as a specifier of which part of the registers do the common names appear in. Moreover, the automaton will operate with a stack belonging to $\mathsf {Sta}'=(\mathbb {C}_{\mathsf {stack}}^\checkmark \times (N_1\rightleftharpoons N_2)\times \mathsf {Reg})^*$. In order to simulate $\mathcal {A}$’s matching conditions for stores, $\mathcal {A}'$ will use, apart from *R*, a symbolic store *S* (which will simulate to the store $\varSigma$ appearing in configurations of $\mathcal {A}$).

For each $(q,R,S)\in Q'$ we include in $\delta '$ precisely the following transitions.

If $q\xrightarrow {\pi _1,\pi _2}q'$ then $(q,R,S)\xrightarrow {(\pi _1\cup \pi _2^+)[\pi ]}(q',R',S')$, where $\pi =\{(j^+,i)\ |\ (i,\pi _2(j),1)\in R,i\notin \mathsf {cod}(\pi _1)\}\cup \{(i,j^+)\ |\ (\pi _1(i),j,2)\in R,j\notin \mathsf {cod}(\pi _2)\}$, $R'=R[\pi _1,\pi _2]$ and $S'=S\circ \overline{\pi }$.^10^ If $q\xrightarrow {\pi _1,\checkmark }q'$ then do the same, with $\pi _2=\mathsf {id}$ (the identity injection). Dually if $q\xrightarrow {\checkmark ,\pi _2}q'$.

If $q\in Q_{P}$ and $q\xrightarrow {\nu X_1.(\ell _1,t_1,\phi _1)^{S_1},\nu X_2.(\ell _2,t_2,\phi _2)^{S_2}}q'$ then if $t_1=t_2$ and for some $R':N_1\rightleftharpoons N_2$:^11^

- $R'=(\mathsf {xdom}(R)\uplus X_1,R\uplus R'',\mathsf {xcod}(R)\uplus X_2)$ where $\mathsf {dom}(R'')=X_1$, $\mathsf {cod}(R'')=X_2$ and for all $(i,j,z)\in R''$ we have $z=1$,
- $\overline{R'}(\ell _1)=\overline{R'}(\ell _2^+)$ and, for all $(i,j,z)\in R'$, $\overline{R'}(S_1(i))=\overline{R'}(S_2(j)^+)$,
- for all $$i\in \mathsf {dom}(S_1){\setminus }\mathsf {dom}(R')$$, $$S_1(i)=S(i)$$,
- for all $$j\in \mathsf {dom}(S_2){\setminus }\mathsf {dom}(R')$$, $$S_2(j)=S(j)$$,

then $$(q,R,S)\xrightarrow {\nu X_1.(\ell ,t_1,\phi )^{S'}}(q',R',S')$$, where:

- $$\ell =\overline{R'}(\ell _1)$$ and $$S'=\overline{R'}(S_1)\cup \overline{R'}(S_2^+)$$,
- if $t_1\in \mathbb {T}_{\mathsf {noop}}$ then $\phi =()$, and if $t_1\in \mathbb {T}_{\mathsf {push}}$ and, say, $\phi _i=(s_i,\pi _i)$ then $\phi =((s_1,s_2,R'[\pi _1,\pi _2]),(\pi _1\cup \pi _2^+)[\pi ])$ with $\pi =\{(i,j^+)\ |\ (\pi _1(i),j,2)\in R',j\notin \mathsf {cod}(\pi _2)\}\cup \{(j^+,i)\ |\ (i,\pi _2(j),1)\in R',i\notin \mathsf {cod}(\pi _1)\}$;

otherwise, if $q\in Q_N$ then switch to divergence mode, i.e. $(q,R,S)\xrightarrow {\mathsf {id}}(\mathsf {div}(q),R,S)$.

If $q\in Q_{P}$ and $q\xrightarrow {\nu X_1.(\ell _1,t_1,\phi _1)^{S_1},\checkmark }q'$ then $(q,R,S)\xrightarrow {\nu X_1.(\ell ,t_1,\phi )^{S'}}(q',S',R')$, where:

- $$\ell =\overline{R'}(\ell _1)$$ and $$S'=\overline{R'}(S_1)$$,
- if $t_1\in \mathbb {T}_{\mathsf {noop}}$ then $\phi =()$, and if $t_1\in \mathbb {T}_{\mathsf {push}}$ and, say, $\phi _1=(s_1,\pi _1)$ then $\phi =((s_1,\checkmark ,R'[\pi _1,\emptyset ]),\pi _1[\pi ])$ with $\pi =\{(i,j^+)\ |\ (\pi _1(i),j,2)\in R'\}$.

Dually if $$q\xrightarrow {\checkmark ,\nu X_2.(\ell _2,t_2,\phi _2)^{S_2}}q'$$.

If $q\in Q_{O}$ and $q\xrightarrow {\nu X_1.(\ell _1,t,\phi _1)^{S_1},\nu X_2.(\ell _2,t,\phi _2)^{S_2}}q'$ then for all $R',R_{\mathsf {p}},\hat{R}:N_1\rightleftharpoons N_2$ and $\pi \in \mathsf {Mix}$ such that:

- if $t\in \mathbb {T}_{\mathsf {noop}}$ then $\hat{R}=R$, and if $t\in \mathbb {T}_{\mathsf {pop}}$ and, say, $\phi _i=(s_i,\pi _i)$ then, assuming that the popped symbol will be $(s_1,s_2,R_{\mathsf {p}})$:
  - $R,\pi _1,\pi _2$ and $R_{\mathsf {p}}$ should be consistent, that is:^12^
    - for each $(i,j,z)\in R$, if $\pi _1(i),\pi _2(j)$ are both defined or $\pi _1(i)\in \mathsf {dom}(R_{\mathsf {p}})$ or $\pi _2(j)\in \mathsf {cod}(R_{\mathsf {p}})$ then $(\pi _1(i),\pi _2(j),z')\in R_{\mathsf {p}}$, for some $z'$,
    - for each $(i,j,z)\in R_{\mathsf {p}}$ if $\pi _1^{-1}(i),\pi _2^{-1}(j)$ are both defined or $\pi _1^{-1}(i)\in \mathsf {dom}(R)$ or $\pi _2^{-1}(j)\in \mathsf {cod}(R)$ then $(\pi _1^{-1}(i),\pi _2^{-1}(j),z')\in R$, for some $z'$;
  - the new pop injection $\pi$ is of the form $\pi =\pi _{A1}\cup \pi _{A2}\cup \pi _{B1}\cup \pi _{B2}$ where:^13^
    - $$\pi _{A1}=\{(\overline{R}(i),\overline{R_{\mathsf {p}}}(\pi _1(i)))\ |\ i\in \mathsf {dom}(\pi _1)\}$$,
    - $$\pi _{A2}=\{(\overline{R}(j^+),\overline{R_{\mathsf {p}}}(\pi _2(j)^+))\ |\ j\in \mathsf {dom}(\pi _2)\}$$,
    - $$\pi _{B1}\subseteq (\mathsf {xdom}(R){\setminus }\mathsf {dom}(R))\times (\mathsf {xcod}(R_{\mathsf {p}}){\setminus }\mathsf {cod}(R_{\mathsf {p}}))^+$$,
    - $$\pi _{B2}\subseteq (\mathsf {xcod}(R){\setminus }\mathsf {cod}(R))^+\times (\mathsf {xdom}(R_{\mathsf {p}}){\setminus }\mathsf {dom}(R_{\mathsf {p}}))$$;
  - the span $\hat{R}$ obtained after popping is then computed by $\mathsf {xdom}(\hat{R})=\mathsf {xdom}(R)\cup (\mathsf {xdom}(R_{\mathsf {p}}){\setminus }\mathsf {cod}(\pi ))$, $\mathsf {xcod}(\hat{R})=\mathsf {xcod}(R)\cup (\mathsf {xcod}(R_{\mathsf {p}}){\setminus }\{j\ |\ j^+\in \mathsf {cod}(\pi )\})$ and $\hat{R}=R\cup R_{A1}\cup R_{A2}\cup R_{A3}\cup R_B$ where:
    - $$R_{A1}=\{(i,j,z)\in R_{\mathsf {p}}\ |\ i\notin \mathsf {cod}(\pi _1),j\notin \mathsf {cod}(\pi _2)\}$$,
    - $$R_{A2}=\{(\pi _1^{-1}(i),j,1)\ |\ (i,j,z)\in R_{\mathsf {p}},i\in \mathsf {cod}(\pi _1),j\notin \mathsf {cod}(\pi _2)\}$$,
    - $$R_{A3}=\{(i,\pi _2^{-1}(j),2)\ |\ (i,j,z)\in R_{\mathsf {p}},i\notin \mathsf {cod}(\pi _1),j\in \mathsf {cod}(\pi _2)\}$$,
    - $$R_B=\{(i,j,1)\ |\ (i,j^+)\in \pi _{B1}\}\cup \{(i,j,2)\ |\ (j^+,i)\in \pi _{B2}\}$$;
- the span $R'$ obtained after the transition satisfies the conditions:
  - $$\mathsf {xdom}(R')=\mathsf {xdom}(\hat{R})\uplus X_1$$ and $$\mathsf {xcod}(R')=\mathsf {xcod}(\hat{R})\uplus X_2$$,
  - $$\hat{R}\subseteq R'$$ and $$R'{\setminus } \hat{R} = R_C\cup R_{C1}\cup R_{C2}$$ where:^14^
    - $$R_C \subseteq X_1\times X_2\times \{1\}$$,
    - $$R_{C1} \subseteq (\mathsf {xdom}(\hat{R}){\setminus }\mathsf {dom}(\hat{R}))\times X_2\times \{1\}$$,
    - $$R_{C2} \subseteq X_1\times (\mathsf {xcod}(\hat{R}){\setminus }\mathsf {cod}(\hat{R}))\times \{2\}$$,
  - $R'(\ell _1)=R'(\ell _2)$ and, for all $(i,j,z)\in R'$, if $S_1(i),S_2(j)$ are both defined then $\overline{R'}(S_1(i))=\overline{R'}(S_2(j)^+)$;

include $$(q,R,S)\xrightarrow {\nu X.(\ell ,t,\phi )^{S'}}(q',R',S')$$, where:

- $$X=(X_1{\setminus }\mathsf {dom}(R_{C2}))\cup (X_2{\setminus }(\mathsf {cod}(R_C)\cup \mathsf {cod}(R_{C1})))^+$$,
- $$\ell =\overline{R'}(\ell _1)$$ and $$S'=\overline{R'}(S_1)\cup \overline{R'}(S_2^+)$$,
- if $t\in \mathbb {T}_{\mathsf {noop}}$ then $\phi =()$, and if $t\in \mathbb {T}_{\mathsf {pop}}$ then $\phi =((s_1,s_2,R_{\mathsf {p}}),\pi )$.

If $q\in Q_{O}$ and $q\xrightarrow {\nu X_1.(\ell _1,t,\phi _1)^{S_1},\checkmark }q'$ then for all $s_2$ and all $R',R_{\mathsf {p}},\hat{R}:N_1\rightleftharpoons N_2$ and $\pi \in \mathsf {Mix}$ such that:^15^

- if $t\in \mathbb {T}_{\mathsf {noop}}$ then $\hat{R}=R$, and if $t\in \mathbb {T}_{\mathsf {pop}}$ and, say, $\phi _1=(s_1,\pi _1)$ then, assuming that the popped symbol will be $(s_1,s_2,R_{\mathsf {p}})$:
  - $R,\pi _1,\pi _2$ and $R_{\mathsf {p}}$ should be consistent, that is, there is no $(i,j,z)\in R$ such that $\pi _1(i)\in \mathsf {dom}(R_{\mathsf {p}})$;
  - the new pop injection $\pi$ is of the form $\pi =\pi _{A1}\cup \pi _{B1}\cup \pi _{B2}$ where:
    - $$\pi _{A1}=\{(\overline{R}(i),\overline{R_{\mathsf {p}}}(\pi _1(i)))\ |\ i\in \mathsf {dom}(\pi _1)\}$$,
    - $$\pi _{B1}\subseteq (\mathsf {xdom}(R){\setminus }\mathsf {dom}(R))\times (\mathsf {xcod}(R_{\mathsf {p}}){\setminus }\mathsf {cod}(R_{\mathsf {p}}))^+$$,
    - $$\pi _{B2}\subseteq (\mathsf {xcod}(R){\setminus }\mathsf {cod}(R))^+\times (\mathsf {xdom}(R_{\mathsf {p}}){\setminus }\mathsf {dom}(R_{\mathsf {p}}))$$;
  - the span $\hat{R}$ obtained after popping is given by $\mathsf {xdom}(\hat{R})=\mathsf {xdom}(R)\cup (\mathsf {xdom}(R_{\mathsf {p}}){\setminus }\mathsf {cod}(\pi ))$, $\mathsf {xcod}(\hat{R})=\mathsf {xcod}(R)\cup (\mathsf {xcod}(R_{\mathsf {p}}){\setminus }\{j\ |\ j^+\in \mathsf {cod}(\pi )\})$ and $\hat{R}=R\cup R_{A1}\cup R_{A2}\cup R_B$ where:
    - $$R_{A1}=\{(i,j,z)\in R_{\mathsf {p}}\ |\ i\notin \mathsf {cod}(\pi _1) \}$$,
    - $$R_{A2}=\{(\pi _1^{-1}(i),j,1)\ |\ (i,j,z)\in R_{\mathsf {p}},i\in \mathsf {cod}(\pi _1) \}$$,
    - $$R_B=\{(i,j,1)\ |\ (i,j^+)\in \pi _{B1}\}\cup \{(i,j,2)\ |\ (j^+,i)\in \pi _{B2}\}$$;
- the span $R'$ obtained after the transition satisfies the conditions:^16^
  - $$\mathsf {xdom}(R')=\mathsf {xdom}(\hat{R})\uplus X_1$$ and $$\mathsf {xcod}(R')=\mathsf {xcod}(\hat{R})$$,
  - $$\hat{R}\subseteq R'$$ and $$R'{\setminus } \hat{R}\subseteq X_1\times (\mathsf {xcod}(\hat{R}){\setminus }\mathsf {cod}(\hat{R}))\times \{2\}$$;

include $$(q,R,S)\xrightarrow {\nu X.(\ell ,t,\phi )^{S'}}(q',R',S')$$, where:

- $$X=X_1{\setminus }\mathsf {dom}(R'{\setminus }\hat{R})$$, $$\ell =\overline{R'}(\ell _1)$$ and $$S'=S[\overline{R'}(S_1)]$$,
- if $t\in \mathbb {T}_{\mathsf {noop}}$ then $\phi =()$, and if $t\in \mathbb {T}_{\mathsf {pop}}$ then $\phi =((s_1,s_2,R_{\mathsf {p}}),\pi )$.

Dually if $$q\xrightarrow {\checkmark ,\nu X_2.(\ell _2,t,\phi _2)^{S_2}}q'$$.

Let us remark that reachable nodes $((q,R,S),\rho ,\sigma ,H)$ in the configuration graph of $\mathcal {A}'$, apart from the initial one, satisfy $\mathsf {dom}(S)=\mathsf {dom}(\rho )=\overline{R}(\mathsf {xdom}(R)\cup \mathsf {xcod}(R))$ while all elements $(s_1,s_2,R,\rho )$ of $\sigma$ also satisfy $\mathsf {dom}(\rho )=\overline{R}(\mathsf {xdom}(R)\cup \mathsf {xcod}(R))$. We arrive at the following.

### Lemma 48

For $\mathcal {A}$ and $\mathcal {A}'$ as above, $\mathcal {A}\sim \mathcal {A}'$.

### Proof

Consider the relation $\mathcal {R}$ between configurations of $\mathcal {A}$ and $\mathcal {A}'$ defined by:

$$\begin{aligned} \mathcal {R}&= \{((q,\rho ,\sigma ,H,\varSigma ),((q,R,S),\rho ',\sigma ',H)) | \rho =\rho '\circ \overline{R},\ \varSigma ='\mathsf {Sto}(\rho ',S),\ \sigma =\mathsf {ev}(\sigma '),\\&\quad \,\, (q,\rho ,\sigma ,H,\varSigma ),((q,R,S),\rho ',\sigma ',H)\text { reachable}\} \end{aligned}$$

where $\mathsf {ev}$ is given by $\mathsf {ev}(\epsilon )=\epsilon$ and $\mathsf {ev}((s_1,s_2,R,\rho )::\sigma )=(s_1,s_2,\rho \circ \overline{R})::\mathsf {ev}(\sigma )$, and by $\varSigma ='\mathsf {Sto}(\rho ',S)$ we mean $\varSigma =S=\emptyset$ or $\varSigma =\mathsf {Sto}(\rho ',S)$. By case analysis we can show that $\mathcal {R}$ is a bisimulation. Moreover, $((q_0,\rho _0,\epsilon ,\emptyset ,\emptyset ),((q_0,R_0,\emptyset ),\rho _0',\epsilon ,\emptyset ))\in \mathcal {R}$, and therefore $\mathcal {A}\sim \mathcal {A}'$. $\square$

In moving from $\mathcal {A}$ to $\mathcal {A}'$, each transition of the former may yield several transitions of the latter, in the case of O- to P-transitions. From these transitions, though, those that have the same label (up to $\sim _\alpha$) are allowed to range solely because of the choice of the final $R'$. In case $\mathcal {A}$ is strongly deterministic, these choices are unique up to $\sim _\alpha$: we make a canonical choice to allocate all common fresh names on the first component of the assignment, while frugality ensures that all fresh names are reachable from those already available, which are uniquely specified from the given *R* and $\hat{R}$. Thus, if $\mathcal {A}$ is strongly deterministic then so is $\mathcal {A}'$.

## Footnotes

1 Two program phrases are regarded as *contextually equivalent*, or simply *equivalent*, if they can be used interchangeably in any context without affecting the observable outcome.

2 A model is *fully abstract* if it captures contextual equivalence denotationally, i.e. equivalence can be confirmed/disproved by reference to the interpretations of terms.

3 Symbolic stores represent stores by use of indices instead of actual names. For example, $S(i)={\texttt {R}}_{j}$ means that in *S* the *i*-th name stores the *j*-th name.

4 Explicitly, for all $i\not =j$, if $\pi (i),\pi (j)$ are both defined then $\pi (i)\not =\pi (j)$.

5 Here, in fact, we refer to the alpha-equivalence relation induced by the $\nu$ binder: for example, $\nu \{1,2\}.({\texttt {R}}_{1},\{(1,{\texttt {R}}_{2}),(2,0),(3,1)\}) \sim _\alpha \nu \{5,6\}.({\texttt {R}}_{6},\{(3,1),(5,0),(6,{\texttt {R}}_{5})\})$.

6 In fact,  is an $(n_0+1,n)$-automaton, but we render it into an $(n_0,n)$ one by changing each $q_1\xrightarrow {\pi }q_2$ to $q_1\xrightarrow {\pi [n_0{+}1\mapsto n_0{+}1]}q_2$.

7 i.e. We stipulate that $\varSigma _1,\varSigma _2$ agree on common names, while values of private names remain unchanged. This stems from the fact that we do not compare plays but, rather, representations thereof.

8 Here we use the alpha-equivalence relation under the $\nu$ binder mentioned in Definition 24.
